# Hypothermia-Mediated Apoptosis and Inflammation Contribute to Antioxidant and Immune Adaption in Freshwater Drum, *Aplodinotus grunniens*

**DOI:** 10.3390/antiox11091657

**Published:** 2022-08-26

**Authors:** Jianxiang Chen, Hongxia Li, Pao Xu, Yongkai Tang, Shenyan Su, Guangxiang Liu, Ningyuan Wu, Miaomiao Xue, Fan Yu, Wenrong Feng, Changyou Song, Haibo Wen

**Affiliations:** 1 Wuxi Fisheries College, Nanjing Agricultural University, Wuxi 214081, China; 2 Key Laboratory of Freshwater Fisheries and Germplasm Resources Utilization, Ministry of Agriculture and Rural Affairs, Freshwater Fisheries Research Center, Chinese Academy of Fishery Sciences, Wuxi 214081, China

**Keywords:** hypothermia, oxidative stress, immunity, inflammation, apoptosis, freshwater drum

## Abstract

Hypothermia-exposure-induced oxidative stress dysregulates cell fate and perturbs cellular homeostasis and function, thereby disturbing fish health. To evaluate the impact of hypothermia on the freshwater drum (*Aplodinotus grunniens*), an 8-day experiment was conducted at 25 °C (control group, Con), 18 °C (LT18), and 10 °C (LT10) for 0 h, 8 h, 1 d, 2 d, and 8 d. Antioxidant and non-specific immune parameters reveal hypothermia induced oxidative stress and immunosuppression. Liver ultrastructure alterations indicate hypothermia induced mitochondrial enlargement, nucleoli aggregation, and lipid droplet accumulation under hypothermia exposure. With the analysis of the transcriptome, differentially expressed genes (DEGs) induced by hypothermia were mainly involved in metabolism, immunity and inflammation, programmed cell death, and disease. Furthermore, the inflammatory response and apoptosis were evoked by hypothermia exposure in different immune organs. Interactively, apoptosis and inflammation in immune organs were correlated with antioxidation and immunity suppression induced by hypothermia exposure. In conclusion, these results suggest hypothermia-induced inflammation and apoptosis, which might be the adaptive mechanism of antioxidation and immunity in the freshwater drum. These findings contribute to helping us better understand how freshwater drum adjust to hypothermia stress.

## 1. Introduction

Water temperature is one of the essential external factors for aquatic animals [1]. Temperature fluctuations beyond the optimum range can trigger a variety of stress reactions in aquatic species, including metabolic problems, physiological malfunction, organ and cell damage, and even death [2,3]. In regard to global climate change and variations in seasonal temperatures, the effect of temperature fluctuations on biological systems is a crucial topic. Due to an increase in climate unpredictability, cold snaps are occurring more frequently and with greater severity [4,5]. Furthermore, the seasonal differences in water temperatures that fish experience during a year might be very substantial. The water temperature in China changes a lot from season to season. For example, the water in Wuxi may get as hot as 30 °C in the summer and as cold as almost 0 °C in the winter. Each year, this has led to a significant economic loss for the aquaculture industry. Therefore, investigating the mechanism of the fish’s responses to low temperatures can contribute to the development of aquaculture.

Since fish are poikilothermic vertebrates, hypothermia exposure can affect their body temperature, which causes a variety of physiological reactions. Hypothermia exposure can result in increases in endogenous reactive oxygen species (ROS) [6]. The imbalance between ROS accumulation and antioxidation defense induces oxidative stress [7]. Oxidative stress leads to physiological disorders, oxidative modification of the biological macromolecules, inflammation, immunosuppression, cellular death including apoptosis, and various pathological conditions as the foundation of many diseases [8,9,10]. Previous studies found that both acute and chronic cold stress could induce oxidative stress in fish tissues [11,12]. It has been reported that acute cold exposure induced high oxidative stress in zebrafish liver, which may lead to mortality [13], and long-term cold exposure also caused oxidative stress in tissues of *Hoplosternum littorale* [14]. Meanwhile, antioxidant defense mechanisms have developed in aquatic animals to cope with hypothermia exposure. The antioxidant enzymes including superoxide dismutase (SOD), catalase (CAT), and glutathione peroxidase (GSH-Px) make up the majority of this system and are crucial for regulating the redox status of cells [15]. Besides the antioxidant system, immune processes are crucial for fish to adjust to hypothermia exposure [16]. Alcorn et al. suggested that the immune apparatus of sockeye salmon reared at 8 °C relied more heavily on the innate immune response [17]. The innate immune system is the first boundary of host defense and plays a vital role in protecting organisms from pathogens and maintaining a physiological steady state under adverse stress conditions [18]. Hypothermia exposure suppresses the innate immune system of fish, thereby increasing susceptibility to infection [19]. Furthermore, many studies have demonstrated that fish tissues have inflammatory reactions as a result of hypothermia [20]. Under hypothermia exposure, inflammatory reactions in tilapia seem tissue specific. The liver showed inconsistent expression of the pro-inflammatory genes such as tumor necrosis factor-α (TNF-α) and interleukin-1 (IL-1β). In addition, hypothermia exposure impairs fish defense mechanisms, causing cellular damage and elevating levels of apoptosis [21]. Apoptosis also plays a key role in the body’s immune response by controlling the number of immune cells, which helps to maintain cellular homeostasis [22].

Freshwater drum (*Aplodinotus grunniens*) is one of the most widely distributed freshwater fish in North and Central America. It is the only species in the genus of *Aplodinotus* that perpetually inhabits freshwater [23]. It is characterized by its abundant nutritional value, scrumptious meat quality, non-intermuscular fishbones, and so on [24]. Therefore, the future of freshwater drum culture seems promising. With these prospects, we imported the freshwater drum larvae from the USA in 2016 and achieved a milestone in artificial farming and breeding in 2019, which provided a breakthrough for aquaculture [24]. According to past studies, freshwater drum is adaptive to water temperatures between 7 and 30 °C [25]. Moreover, as the water temperature dropped to 1 °C or lower, the mortality of juveniles rose [26]. In light of our preliminary research, as a warm-water fish, the optimum temperature range for freshwater drum should be between 18 and 26 °C. However, the winter water temperature in East-Central China is typically below 10 °C. Therefore, this experiment was conducted at 25 °C (as the control group), 18 °C, and 10 °C for 8 days to investigate the mechanism of the hypothermia response of freshwater drum by transcriptomics. To our knowledge, this study is the first to evaluate potential mechanisms of the freshwater drum response to hypothermia exposure based on alterations in antioxidants, non-specific immunity, inflammation, and apoptosis. These studies provide novel insights into how fish respond to hypothermic stress, which could contribute to the risk assessment of hypothermia in the aquatic environment.

## 2. Materials and Methods

### 2.1. Ethics Statement

This study was approved by the Animal Care and Use Committee of Nanjing Agricultural University (Nanjing, China) (WXFC 2021-0006). All animal procedures were performed according to the Guideline for the Care and Use of Laboratory Animals in China.

### 2.2. Experimental Animals and Rearing Conditions

The hypothermia experiment was conducted at Wuxi Fisheries College of Nanjing Agricultural University. Laboratory fish were the first-generation larvae of freshwater drum introduced from the United States by the Freshwater Fisheries Research Center, Chinese Academy of Fishery Sciences. Freshwater drums were reared in chamber temperature-adjustable circulating water systems (specifications for φ 820 × 700 mm) consisting of 12 tanks (300 L each). Freshwater drum averaging 20.88 ± 2.75 g were randomly assigned into 9 tanks (3 tanks per group, 40 fish per tank) and were fed with fresh shrimp (3–5% of their body weight) twice a day (8:00 and 16:00). Prior to the experiment, fish were acclimated in the tanks fed at 25 °C for 30 days. In the experiment, 25 °C was set as the control group (Con), 18 °C, and 10 °C were set as the hypothermia treatments for 8 days. During the experiment, the temperature was gradually decreased from 25 °C to 18 and 10 °C at a rate of 1 °C/h. The room temperature was stabilized at 8 °C. We reduced heat dissipation by covering the mouth of the barrels with cling film. During the 8-day experiment, we cleaned up food scraps and feces daily. Throughout the experiment, dissolved oxygen was kept at >6 mg L^−^^1^, pH 7.2–7.8, and NH_3_ < 0.05 mg L*^−^*^1^.

### 2.3. Sample Collection

Experimental samples were collected at 0 h, 8 h, 1 d, 2 d, 4 d, and 8 d. Fish from each tank were randomly sampled and anesthetized with MS-222 (100 mg L^−1^) at each time point. Blood samples were obtained from the caudal vein and put into anticoagulation tubes. These samples were centrifuged at 5000 rpm at 4 °C for 10 min to extract the plasma. The plasma samples were stored at −80 °C for antioxidant and immune parameter measurements. Meanwhile, the sampled fish were dissected to collect the liver, gut, spleen, head kidney, and caudal kidney on ice, frozen in liquid nitrogen immediately, and stored at −80 °C for subsequent analysis.

### 2.4. Plasma Antioxidant Parameters and Innate Immune Index Analysis

Plasma samples of three fish from each tank were used to measure parameters including total superoxide dismutase (T-SOD), malondialdehyde (MDA), catalase (CAT), glutathione (GSH), glutathione peroxidase (GSH-Px), alkaline phosphatase (AKP), acid phosphatase (ACP), aspartate aminotransferase (AST), and alanine aminotransferase (ALT) according to the manufacturer’s instructions. In detail, T-SOD was determined by the hydroxylamine method (Category No: A001-1-2), MDA was detected by the TBA method (Category No: A003-1-2), GSH was determined by a microplate method (Category No: A006-2-1), GSH-Px was determined by a colorimetric method (Category No: A005-1-2), AKP and ACP were determined by the microenzyme conjugate method (Category No: A059-2 and A060-2), and AST and ALT were determined by the Reitman method (Category No: C010-2-1 and C009-2-1). All the assay kits were purchased from Nanjing Jiancheng Bioengineering Institute, Nanjing, China.

### 2.5. Transmission Electron Microscopy (TEM) Observation

The livers of three fish in each group were cut into small portions (1 mm × 1 mm × 1 mm) separately and then fixed with 2.5% glutaraldehyde for 24 h before transferring to the phosphate buffer (pH 7.4, 4 °C). Then, the tissue portions were fixed in 2% osmium tetroxide in a 0.1 M phosphate buffer (4 °C) for 1.5 h before being dehydrated in ethanol with a series of different concentrations (50, 70, 90, 95, and 100%) for 15 min each and acetone for 15 min. Then, the samples were embedded in epoxy resin (Epoxy Embedding Medium Kit, Sigma) and were cut to ultra-thin sections (70 nm) using the Leica Ultra-cut UCT25 ultramicrotome. Ultra-thin sections were stained with lead citrate and uranyl acetate and then observed using a Hitachi HT7700 transmission electron microscope (Hitachi, Tokyo, Japan). Ten images were saved for each group.

### 2.6. Transcriptome Assembly, Functional Annotation, and Differentially Expressed Genes (DEGs) Analysis

In each group, nine liver tissues were selected to conduct the high-throughput sequence, wherein three fish in each group were randomly mixed and three biological replicates were finally applied for RNA-seq on the Illumina Hiseq6000 platform (Majorbio Bio-pharm Technology Co., Ltd., Shanghai, China). All sequences after quality control generated contig and singleton through de novo assembly were finally connected to obtain transcripts. All transcripts were compared with seven databases including NR (NCBI non-redundant protein sequences), Nt (NCBI non-redundant nucleotide sequences), Pfam (protein families), COG (clusters of orthologous groups of proteins), Swiss-Prot (a manually annotated and reviewed protein sequence database), GO (Gene Ontology), and KEGG (Kyoto Encyclopedia of Genes and Genomes) to obtain functional annotation information. The transcriptome was quantified by RNA-Seq by expectation-maximization (RSEM) to estimate expression abundance. The differentially expressed genes (DEGs) were identified based on the fragments per kilobase of exon model per million mapped reads (FPKM). Because the sequencing depth of samples differs from each other, the absolute gene expression was normalized to the FPKM value, which made the FKPM to be the expression quantity of genes. Deseq2 was used to analyze the variation in DEGs. The criteria for screening DEGs were *p* ≤ 0.05 and a difference in fold change of ≥2 GO and KEGG enrichments were conducted to analyze DEGs.

### 2.7. Validation of Differentially Expressed Genes Obtained from RNA-seq

RNA extraction analysis was conducted according to our previously established methods [27]. Total liver, spleen, gut, head kidney, and caudal kidney RNA of each group were extracted using TRIzol Reagent according to protocols (Takara, Dalian, China). Meanwhile, real-time quantitative PCR (RT-PCR) was also conducted according to our previously established methods [27] to validate the expressions of key genes involved in inflammation and apoptosis. The mRNA sequences for each gene were obtained from the freshwater drum liver transcriptome sequencing database. β-Actin was applied as an internal reference. All the primers were synthesized in Shanghai Generay Biotech Co., Ltd. (Shanghai, China). Details of primers are listed in Table 1. The primers used were designed according to the cds sequences of the genes sequenced from the transcriptome (Table S1 cDNA sequence for the genes refered in manuscript). RT-PCR was performed with SYBR Green (Takara, Dalian, China) on a Takara 800 Fast Real-Time PCR system according to the manufacturer’s protocol.

### 2.8. Correlation Analysis

Pearson’s correlation test was performed to analyze the correlations between parameters or key genes. The significance threshold was set at a *p*-value < 0.05.

### 2.9. Statistical Analysis

The data of immune and antioxidative parameters were analyzed with one-way ANOVA and students’ *t*-test by SPSS 23.0. Relative RNA expression was calculated using the 2^−ΔΔCT^ comparative CT method, one-way ANOVA was applied to compare the statistical difference by SPSS 23.0 (IBM SPSS Statistics, Version 23.0, Armonk, NY, USA).

## 3. Results

### 3.1. Hypothermia Exposure Suppressed Antioxidant and Innate Immunity

**Figure 1:**
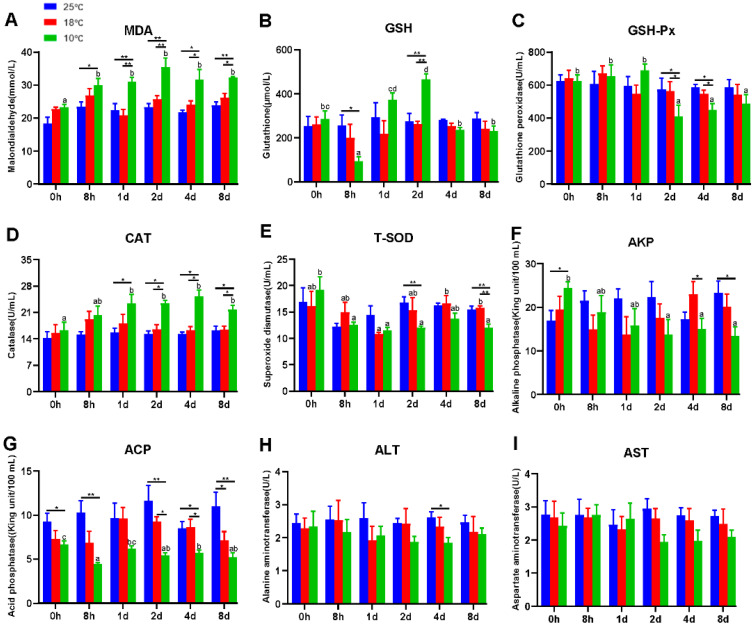
Hypothermia exposure suppressed antioxidant and innate immunity. (**A**), Malondialdehyde, MDA; (**B**), Glutathione, GSH; (**C**), Glutathione peroxidase, GSH-Px; (**D**), Catalase, CAT; (**E**), Total superoxide dismutase, T-SOD; (**F**), Alkaline phosphatase, AKP; (**G**), Acid phosphatase, ACP; (**H**), Alanine aminotransferase, ALT; (**I**), Aspartate aminotransferase, AST. Intra-group data were calculated by one-way ANOVA analysis with SPSS 23.0. Different superscript letters (a, b, c, d) represent the statistical difference inter-group (*p* < 0.05). Inter-group data were calculated by Students’ *t*-test with SPSS 23.0. Asterisks represent the statistical differences inter-group (*, *p* < 0.05; **, *p* < 0.01). Results are expressed as mean ± SEM, *n* = 9.

It is universally acknowledged that hypothermia inevitably induces oxidative stress and immunosuppression. Therefore, antioxidant and innate immune parameters were measured to evaluate the effect of hypothermia exposure on the freshwater drum. In this experiment, antioxidant and innate immune parameters apart from T-SOD exhibited no significant difference at 25 °C and 18 °C with continued exposure (*p* > 0.05) (Figure 1). However, hypothermia at 10 °C dramatically impacted the antioxidant and immune capacity of the freshwater drum. Evidently, the contents of MDA and CAT remarkedly increased with the duration of hypothermia exposure and were significantly higher than the 25 °C and 18 °C groups (*p* < 0.05) (Figure 1A,D). The activity of GSH dramatically declined in the 8 h treatment group (*p* < 0.05), but subsequently elevated significantly reaching a maximum at 2 d (*p* < 0.01), and thereafter decreased to a level that did not differ significantly with the values for the 0 h group (*p* > 0.05) (Figure 1B). In addition, the levels of GSH-Px and T-SOD decreased noticeably at 10 °C and considerably dropped at 2 d in comparison with the 25 °C group (*p* < 0.05) (Figure 1C,E).

In addition, ACP and AKP were found to be significant immunological parameters. As exposure time at 10 °C increased, the levels of AKP and ACP dropped significantly (*p* < 0.05) (Figure 1F,G). Specifically, under 10 °C treatment, AKP activity decreased significantly from 2 d and was significantly lower than the 25 °C group at 8 d (*p* < 0.05) (Figure 1F). Similarly, under the 10 °C treatment, the ACP content was significantly lower than 25 °C and significantly lower than 18 °C from 2 d (*p* < 0.05) (Figure 1G).

The liver is one of the most vulnerable organs to changes in the environment. To further investigate the effect of hypothermia exposure, AST and ALT content, regarded as considerably important indicators of liver function, was measured. Unexpectedly, the activities of ALT and AST did not differ significantly but tended to decrease at 10 °C (*p* > 0.05) (Figure 1H,I).

### 3.2. Morphological Alterations in the Liver of Freshwater Drum Triggered by Hypothermia Exposure

**Figure 2:**
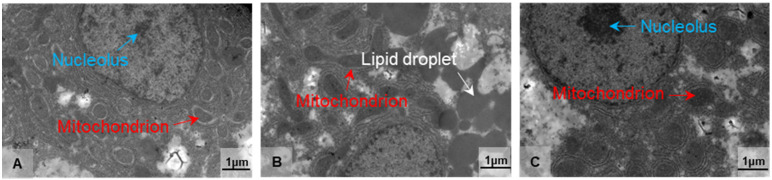
Morphological alterations in the liver of freshwater drum triggered by hypothermia exposure. Morphology and structure of (**A**) Con, (**B**) LT10-2d, and (**C**) LT10-8d, *n* = 3. Arrows in white represent the lipid droplets, in red represent the mitochondria, and in blue represent the nucleolus.

Based on the findings of antioxidant and immunological parameters, we used transmission electron microscopy (TEM) to examine the ultrastructural alterations of the hepatocytes in the treatment groups at 10 °C for 2 and 8 days (LT10-2d and LT10-8d). Compared with the control group, the structure of mitochondria was damaged (Figure 2B). With continuous hypothermic exposure, mitochondria swelled (Figure 2C). In addition, hypothermia induced the accumulation of lipid droplets (Figure 2B), and the aggregation and enlargement of the nucleolus (Figure 2C). These results suggest that hypothermia exposure impaired liver tissue and induced cell fate dysregulation in the liver.

### 3.3. Transcriptome Profiling of DEGs Induced by Hypothermia Exposure in Freshwater Drum

**Figure 3:**
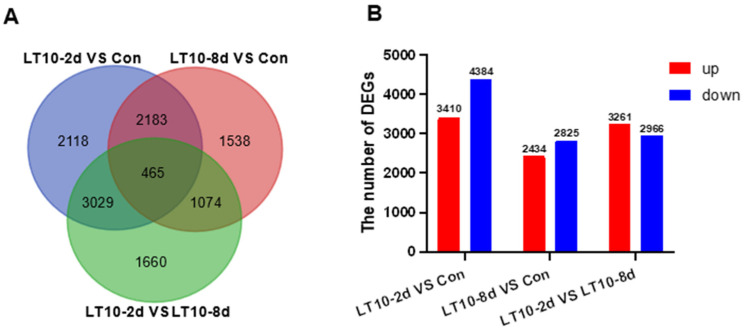
Transcriptome profiling of DEGs induced by hypothermia in freshwater drum. (**A**), Venn diagrams showing the overlap of DEGs among LT10-2d vs. Con (blue), LT10-8d vs. Con (red), and LT10-2d vs. LT10-8d (green). (**B**), Bar chart of DEGs.

To profoundly explain the impact of hypothermia exposure on the molecular mechanism of the liver, the transcriptomes were performed. The differentially expressed genes (DEGs) induced by hypothermia were analyzed by DESeq2 software. According to the sequenced results, DEGs between the control group (Con) and treatment groups (LT10-2d, LT10-8d) were identified. Results of the Venn diagrams displayed only 465 DEGs were overlapped in the pairwise comparison (Figure 3A). With the analysis of transcriptome sequencing, a total of 7794 and 5259 DEGs were identified in LT10-2d and LT10-8d, respectively, of which 3410 and 2434 were upregulated and 4384 and 2825 were downregulated, respectively (Figure 3B). Moreover, a total of 6227 DEGs were counted between LT10-2d and LT10-8d, including 3261 upregulated and 2966 downregulated (Figure 3B).

### 3.4. GO and KEGG Enrichments of DEGs Induced by Hypothermia in Freshwater Drum

**Figure 4:**
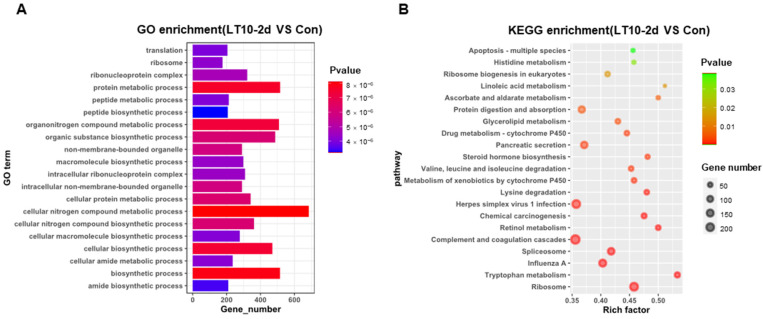

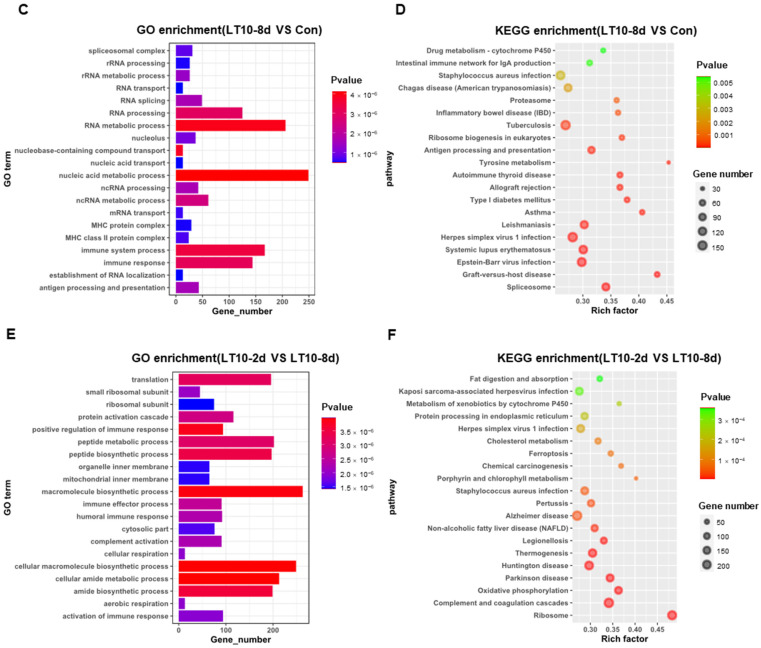
GO and KEGG enrichments of DEGs induced by hypothermia in freshwater drum. (**A**), GO enrichments of DEGs LT10-2d vs. Con; (**B**), KEGG enrichments of DEGs LT10-2d vs. Con; (**C**), GO enrichments of DEGs LT10-8d vs. Con; (**D**), KEGG enrichments of DEGs LT10-8d vs. Con; (**E**), GO enrichments of DEGs LT10-2d vs. LT10-8d; (**F**), KEGG enrichments of DEGs LT10-2d vs. LT10-8d.

In order to better explore the functional relevance of DEGs, GO and KEGG enrichment analyses were performed by corresponding annotations of genes. According to the GO enrichment of LT10-2d, biosynthetic and metabolic processes were the most enriched categories, such as protein metabolic process (GO: 0019538), peptide metabolic process (GO: 0006518), celluar macromolecule biosynthetic process (GO: 0034645) macromolecule biosynthetic process (GO: 0009059), and organic substance biosynthetic process (GO: 1901576) (Figure 4A, Table S2A). Similarly, KEGG enrichment shows that DEGs were mainly enriched in metabolism (tryptophan metabolism, retinol metabolism, lysine degradation, valine, leucine and isoleucine degradation, steroid hormone biosynthesis, glycerolipid metabolism, protein digestion and absorption, ascorbate and aldarate metabolism, and linoleic acid metabolism) and apoptosis (*p* < 0.05) (Figure 4B, Table S3A). However, immunity-related GO terms were significantly enriched in LT10-8d, such as immune system process (GO: 0002376), immune response (GO: 0006955), antigen processing and presentation (GO: 0019882) via MHC protein complex (GO: 0042611), and MHC class II protein complex (GO: 0042613) (*p* < 0.05) (Figure 4C, Table S2B). Meanwhile, according to KEGG enrichment in LT10-8d, the DEGs were mainly involved in metabolism (drug metabolism-cytochrome P450, phenylalanine metabolism, fructose and mannose metabolism, and glycolysis/gluconeogenesis), immunity (antigen processing and presentation, intestinal immune network for IgA production, and Th1 and Th2 cell differentiation), disease (graft-versus-host disease, autoimmune thyroid disease, inflammatory bowel disease (IBD), malaria, and pertussis) (*p* < 0.05) (Figure 4D, Table S3B). Thus, in the comparison of LT10-2d with LT10-8d, the DEGs were mainly enriched in immune- and metabolism-related GO items including humoral immune response (GO: 0006959), immune effector process (GO: 0002252), protein activation cascade (GO: 0072376), peptide metabolic process (GO: 0006518), peptide biosynthetic process (GO: 0043043) (*p* < 0.05) (Figure 4E, Table S2C). Similarly, metabolic- and disease-related pathways were significantly enriched in KEGG including non-alcoholic fatty liver disease (NAFLD), oxidative phosphorylation, fat digestion and absorption, Parkinson disease, Huntington disease, herpes simplex virus 1 infection, Chagas disease (American trypanosomiasis), and long-term depression (*p* < 0.05) (Figure 4F, Table S3C).

### 3.5. Relative Expression of Inflammation-and Apoptosis-Related Genes in Liver Based on RNA-seq under Hypothermia

**Figure 5:**
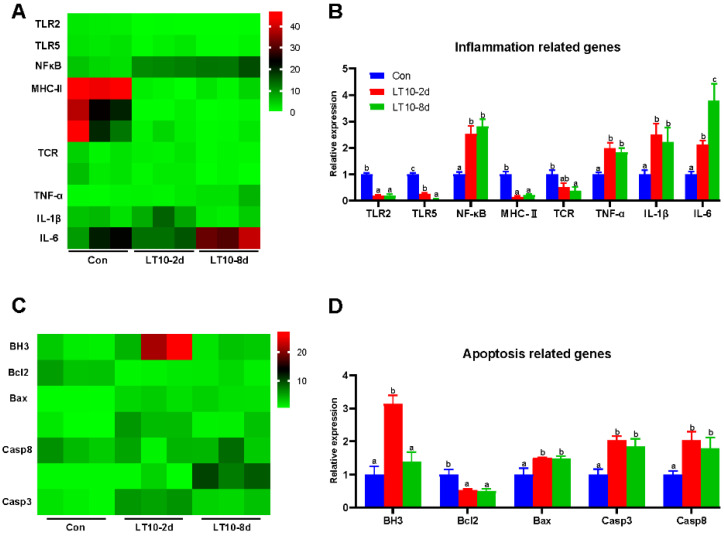
Relative expression of inflammation- and apoptosis-related genes in liver based on RNA-seq under hypothermia. (**A**), Heatmap of expression of inflammation-related genes in transcriptome; (**B**), transcriptional expression of inflammation-related genes in the liver; (**C**), heatmap of expression of apoptosis-related genes in transcriptome; (**D**), transcriptional expression of apoptosis-related genes in the liver. Data were calculated by one-way ANOVA analysis with SPSS 23.0. Different superscript letters (a, b, c) represent the statistical difference (*p* < 0.05). Results are expressed as mean ± SEM, *n* = 9.

Based on the transcriptome results, we selected key genes related to inflammation and apoptosis to be validated in the liver. According to the results of the validation, there was a significant change in gene expression levels regarding inflammation. The gene expression levels of toll-like receptor 2 (TLR2), toll-like receptor (TLR5), major histocompatibility complex II (MHC-II), and TCR (T cell receptor) were significantly downregulated at 10 °C (*p* < 0.05), whereas nuclear factor kappa B (NF-κB), TNF-α, IL-1β, and interleukin-6 (IL-6) were remarkedly upregulated (*p* < 0.05) (Figure 5B). Meanwhile, apoptosis was activated in liver by hypothermia exposure. At 10 °C, the gene expression levels of bcl-2 homolog 3 (BH3), bcl-2 associated X (Bax), caspase 3 (Casp3), and caspase 8 (Casp8) showed remarkable increases (*p* < 0.05), whereas B cell lymphoma-2 (Bcl2) was significantly inhibited at 10 °C (*p* < 0.05) (Figure 5D). Deservedly, the results of validation were consistent with ones in the transcriptome (Figure 5A,C).

### 3.6. Expression of Inflammation-and Apoptosis-Related Genes in Different Immune Organs of the Freshwater Drum under Hypothermia

**Figure 6:**
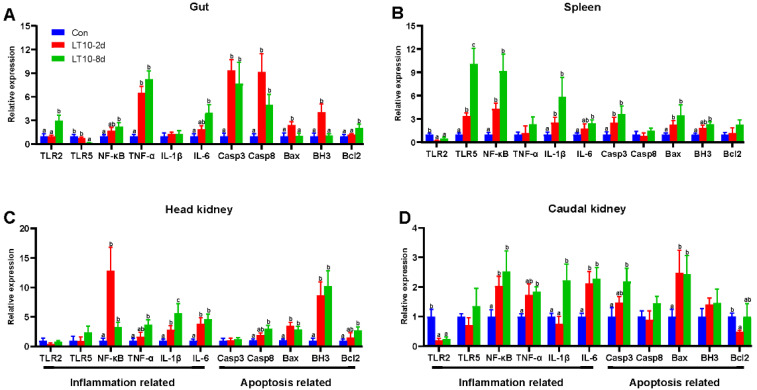
Expression of inflammation- and apoptosis-related genes in different immune organs of the freshwater drum under hypothermia. (**A**), Inflammation- and apoptosis-related genes in the gut; (**B**), inflammation- and apoptosis-related genes in the spleen; (**C**), inflammation- and apoptosis-related genes in the head kidney; (**D**), inflammation- and apoptosis-related genes in the caudal kidney. Data were calculated by one-way ANOVA analysis with SPSS 23.0. Different superscript letters (a, b, c) represent the statistical difference (*p* < 0.05). Results are expressed as mean ± SEM, *n* = 9.

In order to more comprehensively study hypothermia suppressed immunity, inflammation-and apoptosis-related genes were also validated in other immune organs. Although hypothermia induced inflammatory and apoptotic responses in different immune organs, there were differences in the expression of the associated genes. Specifically, in the gut the mRNA levels of inflammation-related genes (TNF-α) and apoptosis-related genes (Casp3, Casp8) were significantly higher than controls under hypothermia exposure (*p* < 0.05) (Figure 6A). In addition, the gene expression of Bax and BH3 were elevated only in the LT10-2d group. (*p* < 0.05) (Figure 6A). Meanwhile, the 8-day hypothermia exposure activated the gene expression of TLR2, NF-κB, IL-6, and Bcl2. (*p* < 0.05) (Figure 6A). However, the mRNA levels of TLR5 decreased rapidly (*p* < 0.05) (Figure 6A).

In the spleen, hypothermia exposure induced a strong upregulation of the mRNA levels of inflammation-related genes (TLR5, NF-κB, IL-1β) and apoptosis-related genes (Casp3 and Bax) compared with Con (*p* < 0.05) (Figure 6B). The gene expression of IL-6 and BH3 was significantly higher than those of the control group with increasing time. (*p* < 0.05) (Figure 6B). Furthermore, surprisingly, the gene expression of TLR2 was significantly suppressed at 10 °C (*p* < 0.05) (Figure 6B).

In the head kidney, the transcript levels of NF-κB, IL-1β, IL-6, Bax, and BH3 were strongly enhanced during hypothermia exposure (*p* < 0.05) (Figure 6C). Additionally, TNF-α, Casp8, and Bcl2 were highly expressed only in LT10-8d (*p* < 0.05) (Figure 6C).

In the caudal kidney, the expression levels of NF-κB, IL-6, and Bax were promoted significantly in LT10-2d and LT10-8d groups (*p* < 0.05) (Figure 6D). Moreover, the mRNA levels of TNF-α, IL-1β, and Casp3 displayed significant upregulation only in LT10-8d (*p* < 0.05) (Figure 6D). However, the gene expression levels of TLR2 and Bcl2 showed a significant decrease under hypothermia exposure.

### 3.7. Apoptosis and Inflammation Were Co-Related with Antioxidant and Immunity under Hypothermia in Freshwater Drum

**Figure 7:**
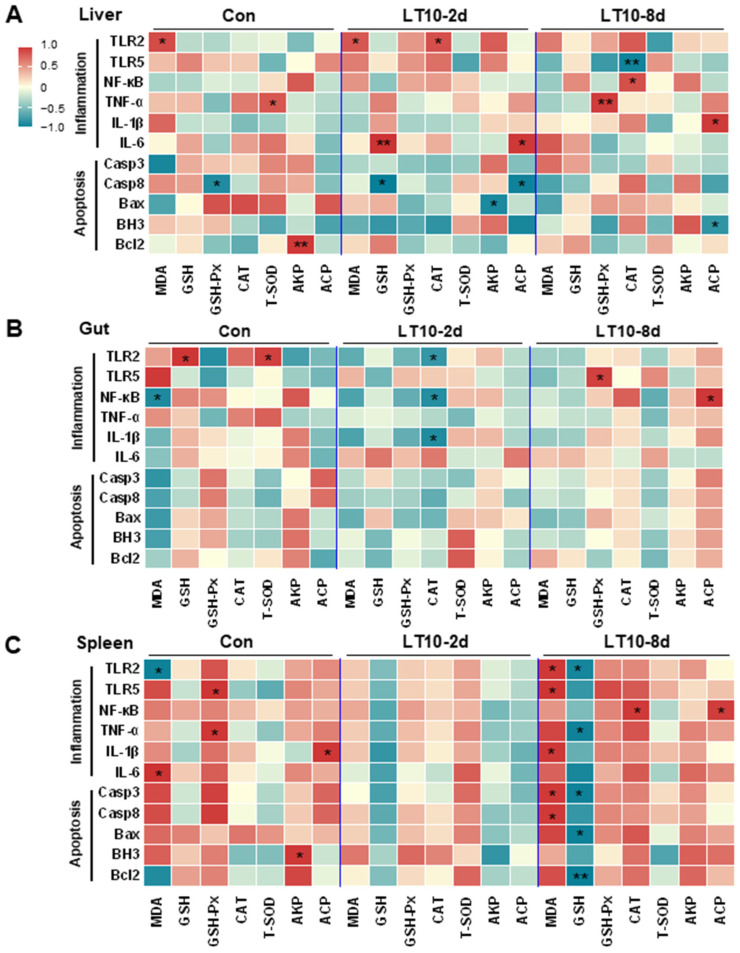

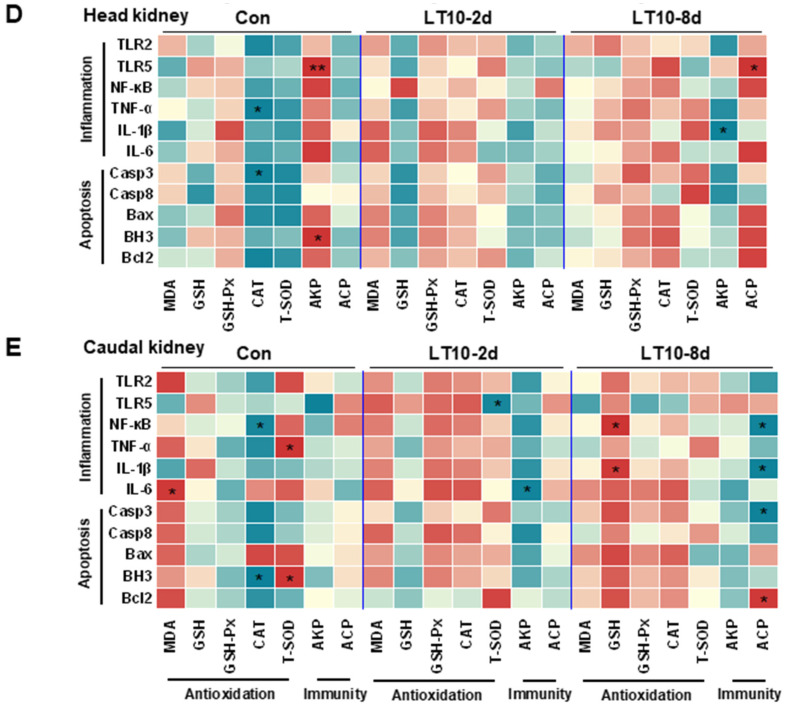
Apoptosis and inflammation were co-related with antioxidant and immunity under hypothermia in freshwater drum. (**A**), Correlation analysis in the liver; (**B**), correlation analysis in the gut; (**C**), correlation analysis in the spleen; (**D**), correlation analysis in the head kidney; and (**E**), correlation analysis in caudal kidney were retrieved from Pearson analysis with SPSS 23.0. * and ** represent the statistical difference (*, *p* < 0.05; **, *p* < 0.01).

Based on the aforementioned information, a Pearson correlation analysis was carried out to investigate the changes brought about by hypothermia exposure. The analysis aimed to better understand the relationship between the expression of inflammation and apoptosis-related genes and antioxidant and innate immune parameters. We discovered that exposure to hypothermia mostly affected the tissues of the liver and spleen (Figure 7A,C), with no discernible association increase seen in the gut, head kidney, or caudal kidney (Figure 7B,D,E). Eight days of hypothermia exposure led to an increase in oxidative stress and inflammation in the liver and spleen tissues (Figure 7A,C). Also, chronic hypothermia induced an increase in the correlation between oxidative stress and apoptosis in the spleen (Figure 7C). In addition, a significant correlation between apoptosis and immunity was found only in the liver under 2 d of hypothermia exposure (Figure 7A).

### 3.8. Hypothermia-Mediated Apoptosis and Inflammation Contributing to Antioxidant and Immune Adaptation in Freshwater Drum

**Figure 8:**
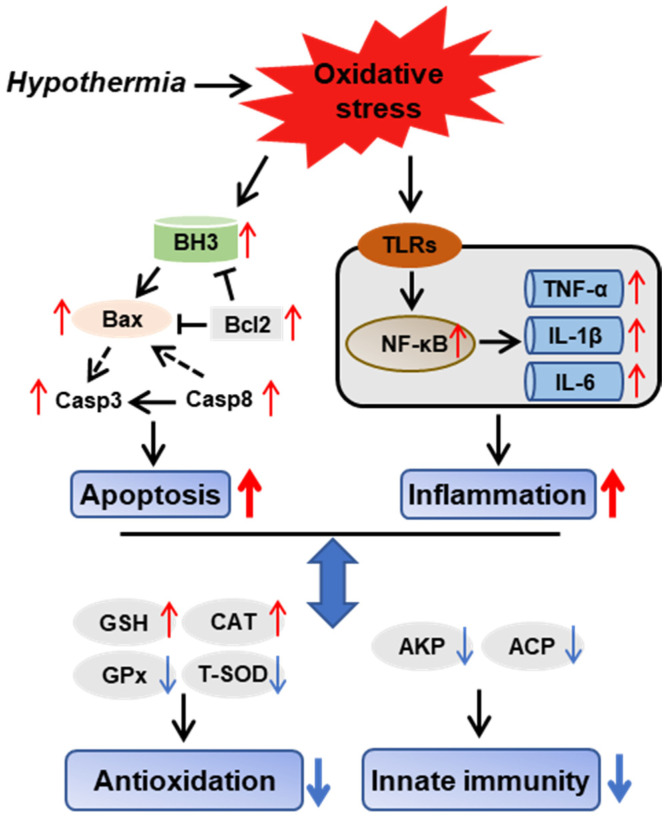
Hypothermia-mediated apoptosis and inflammation contributing to antioxidant and immune adaptation in freshwater drum. Hypothetical regulation of hypothermia on innate immunity, antioxidation, inflammation, and apoptosis was raised based on the results of this study. Solid red arrows represent upregulation. Solid blue arrows represent downregulation. Solid black arrows represent direct impacts, whereas dashed-dotted arrows represent indirect impacts.

We provide a possible schematic of hypothermia reactions in freshwater drum based on the aforementioned findings (Figure 8). The oxidative stress induced by hypothermic exposure impaired antioxidant and immune capacity and triggered inflammation and apoptosis. Meanwhile, there exist relationships between antioxidative, immunological, inflammatory, and apoptotic responses. Hypothermia-mediated inflammation and apoptosis contribute to antioxidant and immune adaption in the freshwater drum.

## 4. Discussion

As one of the most important environmental factors for poikilotherm, changes in water temperature have been investigated to induce a variety of physiological regulation regulations in numerous aquatic species [28,29]. The physiology metabolism process unavoidably leads to the production of reactive oxygen species (ROS) in mitochondria [30]. The excessive production of ROS overwhelms the antioxidant system, which leads to lipid peroxidation resulting in generating a large quantity of MDA [31]. Therefore, MDA content is a biomarker commonly used to assess oxidative stress [32]. In this present study, the MDA content significantly increased with the duration of the hypothermia exposure, suggesting lipid peroxidation was induced on the cytomembrane [33]. Oxidative stress caused by hypothermia exposure may lead to cellular damage, alternations in cell fate, and affects normal physiological functions.

Animals respond to hypothermia-induced oxidative stress through antioxidants. GSH removes ROS in the body, playing an important role in protecting cells from damage [34]. As a peroxidase, GSH-Px can convert peroxides into harmless hydroxy compounds and water to prevent them from oxidizing and forming dangerous free radicals [35]. This study found that the synthesis of GSH increased dramatically in the freshwater drum in the initial phase of hypothermia exposure, indicating that the fish were quickly stressed and the synthesis of GSH increased to remove ROS from the environment [15]. The reduction of GSH content to normal levels at 4 d may be due to the conversion of GSH to oxidized glutathione (GSSG) to reduce GSH-Px to hydrogen peroxide, thereby maintaining the balance of GSH/GSH-Px. Meanwhile, the content of GSH-Px decreased remarkably with prolonged hypothermia exposure. Hypothermia-exposure-induced excess ROS cannot be eliminated by the antioxidant system and, in turn, may reduce enzyme activity. Additionally, antioxidant enzymes CAT and T-SOD are generally considered to be the first line of defense against oxidative stress [36]. T-SOD reduces the superoxide radicals into hydrogen peroxide, which is then decomposed into water and oxygen by CAT. Diverse aquatic species have varying antioxidant capacities when subjected to the same hypothermia exposure. It has been shown that the SOD content of brown noble scallops decreased significantly at 6 h whereas in golden scallops it increased greatly at low temperatures [37]. In the present study, the activity of CAT significantly increased at 10 °C whereas T-SOD decreased markedly, which was similar to the decrease in GSH-Px content. Partly, an antioxidant system would protect the freshwater drum from the oxidative stress induced by hypothermia exposure. However, the production of oxidizing substances that cannot be completely eliminated by the antioxidant system led to the inhibition of antioxidation [38].

In fish, the innate immune system is essential for preserving a complex physiological steady state under stressful circumstances [18]. AKP and ACP are important hydrolases that are involved in intrinsic immune defense and are commonly influenced by environmental stress in fish [39]. The activities of AKP and ACP are indicators to measure immune function and body state, reflecting the defense ability against exogenous microbial infection [40]. Under environmental stress, both the increased and decreased activities of ACP and AKP have been reported in fish [41,42]. In the present study, the levels of AKP and ACP decreased significantly under hypothermia exposure, suggesting hypothermia exposure suppressed the immune response and weakened disease resistance in the freshwater drum.

The liver is an important organ of metabolism and immunity in aquatic animals [43] and it is sensitive to external environmental changes, accompanied by ultrastructural alterations [44]. The nucleus, which is the largest and most significant component of eukaryotic cells, serves as the primary location for the storage, replication, and transcription of genetic material within the cell. By preserving gene integrity and managing cellular processes, it controls gene expression [45]. The mitochondrion is the dominant organelle to produce energy and is concurrently involved in cell differentiation, cell messaging, apoptosis, and cell growth and cycle regulation. In the present study, there were slight distortions in mitochondria and nuclei under hypothermia exposure, which were consistent with the results of Collins [46]. ROS produced non-specific damage to lipids, proteins, and DNA, resulting in altered cell destiny, as evidenced by nucleus damage and mitochondrial swelling [47]. Apoptosis-related gene expression provides proof of this. In addition, mitochondria dysmorphology might be the reason for the increase in the number of lipid droplets. Mitochondrial abnormality inhibited lipid decomposition and impacts fatty acid β-oxidation. As a consequence, lipid droplets in the liver accumulated under hypothermia exposure. Furthermore, some studies indicate that lipid droplets are associated closely with inflammatory responses [48]. These alterations on organelles led to cellular damage and inevitably affected the normal function of the liver. Meanwhile, the variations in AST and ALT levels served as additional support for this. In this investigation, the levels of AST and ALT tended to decrease at 10 °C, demonstrating that exposure to hypothermia suppressed hepatic function.

Transcriptomics refers to a discipline on gene transcription and regulation in cells that analyzes gene expression at the RNA level. The utilization of transcriptomics will reveal the hypothermia response mechanism on freshwater drum more comprehensively and profoundly. In the present study, the DEGs were mainly involved in metabolism, immunity, inflammation, programmed cell death, and disease. It is well known that the regulation of metabolism is an important part of the temperature response in fish [49]. Hypothermia responses consume a lot of energy, therefore, the DEGs were significantly enriched in protein digestion and absorption, glycolysis/gluconeogenesis, and fat digestion and absorption. Hypothermia exposure promoted oxidative stress in a chronic manner. Persisting oxidative stress caused damage to substances in cells such as lipids and proteins and finally led to apoptosis in the freshwater drum [50]. Furthermore, hypothermia exposure limited antigen processing and presentation and affected Th1 and Th2 cell differentiation synchronously, thereby causing immunosuppression in the body [51]. In addition, oxidative stress activated a large range of inflammation-related transcription factors. Subsequently, these transcription factors induced the expression of many cytokines and chemokines, leading to chronic inflammation in the liver under hypothermia exposure. Finally, with the accumulation of various adverse responses to hypothermia exposure, the outbreak of endogenous and exogenous diseases in the freshwater drum may affect the body health and even cause death.

Immune organs play an important role in producing or storing immune cells. In fish, the organs of the immune system include the gills, thymus, spleen, head kidney, caudal kidney, liver, and gut [52,53]. However, there are relatively few studies on the effects of hypothermia exposure on immune organs. This study investigated the effects of oxidative stress induced by hypothermia exposure on immune organs from the perspective of inflammatory and apoptotic responses. The inflammatory response is a common consequence in fish under adverse environments, which usually was associated with oxidative stress. In recent years, accumulating evidence highlighted the role of TLRs/NF-κB signaling in inflammation caused by the antioxidative response, including the upstream and the downstream regulators [54,55]. As a specific pathogen recognition receptor, TLRs are regarded as pivotal regulators linking oxidative stress to inflammation [56,57] that can induce activation of NF-κB. In addition, MHC–peptide complexes play an important role in adaptive immunity. MHC-II is mainly involved in the presentation of exogenous antigens and is capable of endogenous antigen presentation under some conditions [58]. The treated antigen fragment is presented to TCRs during the initial phase of the immune response [59], which could eventually exhibit inflammatory functions via NF-κB signaling [60]. Furthermore, activation of NF-κB induces gene expression of inflammatory factors such as TNF-α, IL-1 β, and IL-6 [61]. In the present study, upregulated gene expression of NF-κB and inflammatory factors indicate hypothermia exposure promoted inflammatory responses in different organs. However, gene expression of TLRs in different organs showed different trends, suggesting the inflammatory response to hypothermia exposure showed a tissue-specific difference [15]. The underlying reasons for this are unclear. We hypothesized that TLR-mediated signal transmission may be independent of NF-κB. Moreover, mRNA levels of MHC-II and TCR in the liver were suppressed under hypothermia exposure. The suppression of immunity by low temperature was confirmed again. Meanwhile, correlation analysis reveals the closer association between antioxidative response and inflammatory response under hypothermia exposure was enhanced in the liver and spleen. Inflammation caused by oxidative stress was further confirmed. The liver and spleen may be the important target organs for inflammatory responses induced by oxidative stress under hypothermia exposure. Apoptosis can remove the unnecessary or abnormal cells in multicellular organisms to maintain normal physiological functions. In the extrinsic pathway, caspase-8 can cause the following cascade reaction. In the intrinsic pathway, Bcl-2 and Bax mediate caspase-9. Activated caspase-9 further initiates caspase-3 to accomplish apoptosis. In this study, apoptosis-related genes were activated in different organs, indicating apoptosis was the extensive and key response mechanism to maintain homeostasis under hypothermia exposure and further demonstrated the molecular mechanism induced by hypothermia in the freshwater drum. Through correlation analysis among antioxidants, immunity, and apoptosis, we found there was a significant correlation between antioxidants and apoptosis in the spleen during hypothermia treatment, suggesting apoptosis was associated with oxidative stress. Inhibition of antioxidant capacity might promote the apoptotic response [62,63]. Meanwhile, the correlation between immunity and apoptosis in liver was also found. The accelerated apoptosis induced by hypothermia exposure can control the death of immune cells in immune organs and thereby affects the immune function in the body.

## 5. Conclusions

In conclusion, hypothermia induced oxidative stress and suppressed antioxidant and innate immunity in the freshwater drum. Inflammation and apoptosis were activated in response to hypothermia exposure, which might contribute to the antioxidant and innate immunity adaption. These alternations reveal the underlying mechanisms of hypothermia responses in the freshwater drum.

## Figures and Tables

**Table 1:** Primers and sequences referred to in the experiment.

	Primer	Sequence (5′ → 3′)	Amplification Size (bp)
TLR5	F	CGACCTCGGAGCCCAGAAT	139
	R	CAAACCAGACAATCCCACAA	
TLR2	F	GGAGAAACCAGTGGGTCAAG	103
	R	CAACAGAATGGCGACAAATAG	
TCR	F	ATCTTCCGTCTTCCAAACCA	182
	R	AGCCATTCACTCACTGCCTC	
NF-κB	F	GTGGGAGGAGGAGTTTGA	113
	R	TATCGCAGCCCATCTATG	
MHC-II	F	ATCAGCTTCTCCCTCCTCTTC	179
	R	AATCCAACAAACTTTCCCACA	
TNF-α	F	ATCGGCGTGCTGTTCAA	219
	R	GCGACCGTGGGATTTAG	
IL-1	F	GTTCTCGGCGTCTGATTGTG	193
	R	GTGAGGCGGTGCTGGTGTTC	
IL-6	F	GCCCAGGGAAGCCTGAGAAT	112
	R	GGGACATCCGTGGTTTGACG	
BH3	F	CCCAAGACGGGTTGTGAT	125
	R	CCATATTGCCCTGCAAGTAG	
Casp8	F	GGAGAACCGACTGGAGGAA	134
	R	TGTAGATGGAGCCTGTGGAAG	
Bcl2	F	CGGGTCAATAGTTGCTCCTC	224
	R	CCGTGGTGGAGGTGAGATAC	
Bax	F	GAGGTGGTGGAACATCTGCT	209
	R	TTGGTGGTCAGTGCCTTGTA	
Casp3	F	CTGCTACGCCTCGTTTGTCT	240
	R	TCAGCTTCCACAGGGATCTT	
β-Actin	F	AAATCGTGCGTGGACATCA	127
	R	CCGTCAGGGCAGCTCATAG	

Note: The mRNA sequences for each gene were obtained from the freshwater drum liver transcriptome sequencing database which was preserved in the lab. The primers used were designed according to the cds sequences of the genes sequenced from the transcriptome (Table S1). Primers for RT-PCR were designed using primer premier 5.0.

## Data Availability

The data are contained within the article and Supplementary Materials.

## Supplementary Materials

The following supporting information can be downloaded at: https://www.mdpi.com/article/10.3390/antiox11091657/s1, Table S1: cDNA sequence for the genes referred in the experiment; Table S2: GO enrichment analysis statistical table; Table S3: KEGG enrichment analysis statistical table.

## References

[1] Song C., Cui Y., Liu B., Xie J., Ge X., Xu P., Lin Y. (2018). HSP60 and HSP90β from blunt snout bream, *Megalobrama amblycephala*: Molecular cloning, characterization, and comparative response to intermittent thermal stress and *Aeromonas hydrophila* infection. Fish Shellfish. Immunol..

[2] Snyder R.J., Hennessey T.M. (2003). Cold tolerance and homeoviscous adaptation in freshwater alewives (*Alosa pseudoharengus*). Fish Physiol. Biochem..

[3] Song C., Liu B., Jiang S., Xiong Y., Sun C., Zhou Q., Jiang Z., Liu B., Zhang H. (2020). Anthraquinone extract from *Rheum officinale* Bail improves growth performance and Toll–Relish signaling-regulated immunity and hyperthermia tolerance in freshwater prawn *Macrobrachium nipponense*. 3 Biotech..

[4] Tingley M.P., Huybers P. (2013). Recent temperature extremes at high northern latitudes unprecedented in the past 600 years. Nature.

[5] Pirhalla D.E., Sheridan S.C., Ransibrahmanakul V., Lee C.C. (2015). Assessing cold-snap and mortality events in south Florida coastal ecosystems: Development of a biological cold stress index using satellite SST and weather pattern forcing. Estuar. Coast..

[6] Blagojevic D.P., Grubor-Lajsic G.N., Spasic M.B. (2011). Cold defence responses: The role of oxidative stress. Front. Biosci..

[7] Newsholme P., Cruzat V.F., Keane K.N., Carlessi R., Bittencourt P.D. (2016). Molecular mechanisms of Ros production and oxidative stress in diabetes. Biochem. J..

[8] Song C., Liu B., Ge X., Li H., Liu B., Xu P. (2022). miR-34a/Notch1b mediated autophagy and apoptosis contributes to oxidative stress amelioration by emodin in the intestine of teleost *Megalobrama amblycephala*. Aquaculture.

[9] Sindhi V., Gupta V., Sharma K., Bhatnagar S., Kumari R., Dhaka N. (2013). Potential applications of antioxidants—A review. J. Pharm. Res..

[10] Apak R., Özyürek M., Guclu K., Capanoglu E. (2016). Antioxidant activity/capacity measurement. III. reactive oxygen and nitrogen species (Ros/Rns) scavenging assays, oxidative stress biomarkers, and chromatographic/chemometric assays. J. Agric. Food Chem..

[11] Vinagre C., Madeira D., Narciso L., Cabral H.N., Diniz M. (2012). Effect of temperature on oxidative stress in fish: Lipid peroxidation and catalase activity in the muscle of juvenile seabass, Dicentrarchus labrax. Ecol. Indic..

[12] Tseng Y.C., Chen R.D., Lucassen M., Schmidt M.M., Dringen R., Abele D., Hwang P.P. (2011). Exploring uncoupling proteins and antioxidant mechanisms under acute cold exposure in brains of fish. PLoS ONE.

[13] Lu D., Ma Q., Sun S., Zhang H., Chen L., Zhang M., Du Z. (2019). Reduced oxidative stress increases acute cold stress tolerance in zebrafish. Comp. Biochem. Pysiol. A Mol. Integr. Physiol..

[14] Rossi A., Bacchetta C., Cazenave J. (2017). Effect of thermal stress on metabolic and oxidative stress biomarkers of *Hoplosternum littorale* (*Teleostei, Callichthyidae*). Ecol. Indic..

[15] Chen Y., Liu E., Li C., Pan C., Zhao X., Wang Y., Ling Q. (2020). Effects of heat stress on histopathology, antioxidant enzymes, and transcriptomic profiles in gills of pikeperch *Sander lucioperca*. Aquaculture.

[16] Cui Y., Hou Z., Ren Y., Men X., Zheng B., Liu P., Xia B. (2020). Effects of aerial exposure on oxidative stress, antioxidant and non-specific immune responses of juvenile sea cucumber *Apostichopus japonicus* under low temperature. Fish Shellfish. Immunol..

[17] Alcorn S., Murray A., Pascho R. (2002). Effects of rearing temperature on immune functions in sockeye salmon (*Oncorhynchus nerka*). Fish Shellfish. Immun..

[18] Magnadóttir B. (2006). Innate immunity of fish (overview). Fish Shellfish. Immunol..

[19] Song H., Xu D., Tian L., Chen R., Wang L., Tan P., You Q. (2019). Overwinter mortality in yellow drum (*Nibea albiflora*): Insights from growth and immune responses to cold and starvation stress. Fish Shellfish Immun..

[20] Dellagostin E.N., Martins A.W.S., Blödorn E.B., Silveira T.L.R., Komninou E.R., Junior A.S.V., Corcini C.D., Nunes L.S., Remião M.H., Collares G.L. (2022). Chronic cold exposure modulates genes related to feeding and immune system in Nile tilapia (*Oreochromis niloticus*). Fish Shellfish Immun..

[21] Cheng C., Ye C., Guo Z., Wang A. (2017). Immune and physiological responses of pufferfish (*Takifugu obscurus*) under cold stress. Fish Shellfish Immun..

[22] Miest J.J. (2013). Apoptosis and Its Association with Immunomodulation and Disease in Common Carp (*Cyprinus carpio* L.). Ph.D. Dissertation.

[23] Hernández-Gómez R.E., Contreras-Sánchez W.M., Hernández-Franyutti A., Perera-García M.A., Torres-Martínez A. (2021). Testicular structure and development of the male germinal epithelium in the freshwater drum *Aplodinotus grunniens* (*Perci-formes: Sciaenidae*) from the Usumacinta River, Southern Mexico. Acta Zool..

[24] Song C., Wen H., Liu G., Ma X., Lv G., Wu N., Chen J., Xue M., Li H., Xu P. (2022). Gut Microbes Reveal Pseudomonas Medicates Ingestion Preference via Protein Utilization and Cellular Homeostasis Under Feed Domestication in Freshwater Drum, *Aplodinotus grunniens*. Front. Microbiol..

[25] William B.W. (1968). Life history aspects of smallmouth buffalo and freshwater drum in wheeler reservoir, Alabama. Proc. Annu. Conf. Southeast. Assoc. Game Fish Comm..

[26] Bodensteiner L.S., Lewis W.M. (1992). Role of Temperature, Dissolved Oxygen, and Backwaters in the Winter Survival of Freshwater Drum (Aplodinotus grunniens) in the Mississippi River. Can. J. Fish. Aquat. Sci..

[27] Li H., Qiang J., Song C., Xu P. (2020). Transcriptome profiling reveal *Acanthopanax senticosus* improves growth performance, immunity and antioxidant capacity by regulating lipid metabolism in GIFT (*Oreochromis niloticus*). Comp. Biochem. Physiol. Part D Genom. Proteom..

[28] Jiang S., Zhou F., Yang Q., Huang J., Yang L., Jiang S. (2019). Impact of temperature stress on oxygen and energy metabolism in the hepatopancreas of the black tiger Shrimp, *Penaeus monodon (Crustacea: Decapoda: Penaeidae*). Pak. J. Zool..

[29] Fan L., Wang L., Wang Z. (2019). Proteomic characterization of the hepatopancreas in the Pacific white shrimp *Litopenaeus vannamei* under cold stress: Revealing the organism homeostasis mechanism. Fish Shellfish. Immunol..

[30] Apel K., Hirt H. (2004). Reactive oxygen species: Metabolism, oxidative stress, and signal transduction. Annu. Rev. Plant Biol..

[31] Lykkesfeldt J., Svendsen O. (2007). Oxidants and antioxidants in disease: Oxidative stress in farm animals. Vet. J..

[32] Tsikas D. (2017). Assessment of lipid peroxidation by measuring malondialdehyde (MDA) and relatives in biological samples: Analytical and biological challenges. Analytical. Biochem..

[33] Géret F., Jouan A., Turpin V., Bebianno M.J., Cosson R.P. (2002). Influence of metal exposure on metallothionein synthesis and lipid peroxidation in two bivalve mollusks: The oyster (*Crassostrea gigas*) and the mussel (*Mytilus edulis*). Aquat. Living Resour..

[34] Kosower N.S., Kosower E.M. (1978). The glutathione status of cells. Int. Rev. Cytol..

[35] Imai H., Nakagawa Y. (2003). Biological significance of phospholipid hydroperoxide glutathione peroxidase (PHGPx, GPx4) in mammalian cells. Free. Radic. Biol. Med..

[36] Ojha A., Yaduvanshi S.K., Srivastava N. (2010). Effect of combined exposure of commonly used organophosphate pesticides on lipid peroxidation and antioxidant enzymes in rat tissues. Pestic. Biochem. Physiol..

[37] Tan K., Zhang B., Ma H., Li S., Zheng H. (2019). Oxidative stress responses of golden and brown noble scallops *Chlamys nobilis* to acute cold stress. Fish Shellfish Immun..

[38] Wei H., Zhang R., Su Y., Bi Y., Li X., Zhang X., Bao J. (2018). Effects of Acute Cold Stress After Long-Term Cold Stimulation on Antioxidant Status, Heat Shock Proteins, Inflammation and Immune Cytokines in Broiler Heart. Front. Physiol..

[39] Hong Y., Huang Y., Yan G., Pan C., Zhang J. (2018). Antioxidative status, immunological responses, and heat shock protein expression in hepatopancreas of Chinese mitten crab, *Eriocheir sinensis* under the exposure of glyphosate. Fish Shellfish. Immunol..

[40] Broeg K. (2010). The activity of macrophage aggregates in the liver of flounder (*Platichthys flesus*) and wrasse (*Symphodus melops*) is associated with tissue damage. Mar. Environ. Res..

[41] Jia R., Du J., Cao L., Feng W., He Q., Xu P., Yin G. (2020). Immune, inflammatory, autophagic and DNA damage responses to long-term H_2_O_2_ exposure in different tissues of common carp (*Cyprinus carpio*). Sci. Total Environ..

[42] Majumder R., Kaviraj A. (2019). Acute and sublethal effects of organophosphate insecticide chlorpyrifos on freshwater fish *Oreochromis niloticus*. Drug Chem. Toxicol..

[43] Meszaros A., Weidinger A., Dumitrescu S., Müllebner A.V., Duvigneau J.C., Kozlov A.V. (2017). The Impact of Pro-inflammatory Cytokines on ROS Mediated Liver Damage. Free. Radic. Biol. Med..

[44] Xu Z., Regenstein J.M., Xie D., Lu W., Ren X., Yuan J., Mao L. (2018). The oxidative stress and antioxidant responses of *Litopenaeus vannamei* to low temperature and air exposure. Fish Shellfish. Immunol..

[45] Shen W., Balajee A., Wang J., Wu H., Eng C., Pandolfi P.P., Yin Y. (2007). Essential Role for Nuclear PTEN in Maintaining Chromosomal Integrity. Cell.

[46] Collins P. (2010). Environmental stress upon hepatopancreatic cells of freshwater prawns (*Decapoda: Caridea*) from the floodplain of Paraná River. Nat. Sci..

[47] Zhao K., Zhao G., Wu D., Soong Y., Birk A.V., Schiller P.W., Szeto H.H. (2004). Cell-permeable Peptide Antioxidants Targeted to Inner Mitochondrial Membrane inhibit Mitochondrial Swelling, Oxidative Cell Death, and Reperfusion Injury. J. Biol. Chem..

[48] Melo R.C.N., Dvorak A.M. (2012). Lipid Body-Phagosome Interaction in Macrophages during Infectious Diseases: Host Defense or Pathogen Survival Strategy?. PLoS Pathog..

[49] Vornanen M., Hassinen M., Koskinen H., Krasnov A. (2005). Steady-state effects of temperature acclimation on the transcriptome of the rainbow trout heart. Am. J. Physiol. Regul. Integr. Comp. Physiol..

[50] Liao Z., Lin D., Jia J., Cai R., Yu Y., Li W. (2021). Innate Immune Response to Fasting and Refeeding in the Zebrafish Kidney. Biomolecules.

[51] Gein S.V., Sharav’eva I.L. (2018). Immunomodulating Effects of Cold Stress. Biol. Bull. Rev..

[52] Whyte S.K. (2007). The innate immune response of finfish—A review of current knowledge. Fish Shellfish. Immunol..

[53] Novoa B., Figueras A. (2012). Zebrafish: Model for the Study of Inflammation and the Innate Immune Response to Infectious Diseases. Curr. Top. Innate Immun. II.

[54] Kane L.P., Shapiro V.S., Stokoe D., Weiss A. (1999). Induction of NF-kappa B by the Akt PKB kinase. Curr. Biol..

[55] Thome M., Tschopp J. (2003). TCR-induced NF-κB activation: A crucial role for Carma1, Bcl10 and MALT1. Trends Immunol..

[56] West A.P., Brodsky I.E., Rahner C., Woo D.K., Erdjument-Bromage H., Tempst P., Ghosh S. (2011). TLR signaling augments macrophage bactericidal activity through mitochondrial ROS. Nature.

[57] Jiang X., Fang L., Wu H., Mei X., He F., Ding P., Liu R. (2017). TLR2 Regulates Allergic Airway Inflammation and Autophagy Through PI3K/Akt Signaling Pathway. Inflammation.

[58] Dengjel J., Schoor O., Fischer R., Reich M., Kraus M., Müller M., Stevanovic S. (2005). Autophagy promotes MHC class II presentation of peptides from intracellular source proteins. Proc. Natl. Acad. Sci. USA.

[59] Birnbaum M.E., Mendoza J.L., Sethi D.K., Dong S., Glanville J., Dobbins J., Garcia K.C. (2014). Deconstructing the peptide-MHC specificity of t cell recognition. Cell.

[60] dos Santos N.R., Ghezzo M.N., da Silva R.C., Fernandes M.T. (2010). NF-κB in T-cell acute lymphoblastic leukemia: Oncogenic functions in leukemic and in microenvironmental cells. Cancers.

[61] Gribar S.C., Anand R.J., Sodhi C.P., Hackam D.J. (2008). The role of epithelial toll-like receptor signaling in the pathogenesis of intestinal inflammation. J. Leukoc. Biol..

[62] Murakawa M., Jung S.-K., Lijima K., Yonehara S. (2001). Apoptosis-inducing protein, AIP, from parasite-infected fish induces apoptosis in mammalian cells by two different molecular mechanisms. Cell Death Differ..

[63] Woo A.H., Park L., Park M., Lee H., Lee S., Chun Y., Lee S., Hong S., Rhee C.H. (2002). Arsenic trioxide induces apoptosis through a reactive oxygen species-dependent pathway and loss of mitochondrial membrane potential in HeLa cells. Int. J. Oncol..

