# Supplementary material for: Hypothermia-Mediated Apoptosis and Inflammation Contribute to Antioxidant and Immune Adaption in Freshwater Drum, Aplodinotus grunniens
Source: Antioxidants (Basel). 2022 Aug 26;11(9):1657. doi: 10.3390/antiox11091657 (PMC9495763; doi:10.3390/antiox11091657)
Supplement: Supplementary file 1 [file antioxidants-11-01657-s001.zip › Table. S1 cDNA sequence for the genes refered in manuscript(antioxidants-1848579).pdf]

TLR5
Transcript ID: transcript4942_f3p0_3143
Annotation in NR: soluble toll-like receptor 5 [Miichthys miiuy] KR709249.1

ATGTGGACACTGAGTCTTCAGGCGGTTGTCATCTGTGTTTTCCTACAGGTGCCGGGGTGTT
TCCCATCATGCATCATAATGGGCTCTGTAGCCAGCTGTGCCTACCAAAACCTCCGCTGGAT
TCCTCCTCTCCCTCCCAACATCACCCACCTGTACCTGGAGATGAACCGCATTGGTGAGATC
AACTCCACCTCCCTGTCAGGCTTGGAGGAGCTACAGGAGCTCGACCTCGGAGCCCAGAA
TGTACCGCTTGTAATCAAGAACAACTCGTTCAGCGGTCAAAGACGTCTGAAGAGGCTGG
TGCTCAACGCCAACGTTGGCCTTCGTCTGGAGCCAAAGGCTTTTGTGGGATTGTCTGGTTT
GCGGAATCTCTACCTGGGTTACTGTTTGCTTAAGGAGTCCATCCTGAAGGAGAACTATCTA
GAGCCGCTGTCCTCCTTAGAGACTCTTGACCTGTTTGGTAACAACATAAACAAGCTGCAG
CCTGCAATGTTCTTTGTGAACATGACTAATTTGAAAGTTTTGAATCTCAAGTTGAACACAT
TTGACAAAATATGTGAGTCTGATCTTGTTGGTTTCCAGGGAAAGCACTTTGAGAAACTGA
ACTTGAACTCTGTTCACTTTAGGGATATGTCTAAAACATCTTTTGACTGGGAGAAATGTGG
GAACCCTTTTAGAGGAATGTCCTTTCAGTCACTCGACCTGTCCTACAATGGGTTAAGTTTG
GGTAAATCAAAGCAGTTTTTCAGAGCCATCGAGGGGACTAAGATCTCCCATCTTAAAATG
TCAGGACACATCGGTAAAGGATTTTCATTCAGCAATCTCCCTGATCCAGACCGCAACACG
TTTGAAGGCCTGACAAACAGTTTGATCCACACTTTGGATCTGTCTAAAAACAGGATATTTG
CATTGCAACAGGGGGTTTTTAGTCCGTTGAAAAAGGCTGCAATTATTGACATTTCCCAAA
ACCAGGTTAATGAGATACACAGAAATGCCTTTGAAGGTCTTCAGGGACATTTGAAAATGC
TCAACCTGTCACGCAACCTGCTGGGGGAAATCTATTCTCACACTTTTGCTTCTCTGACAAA
CCTGAAAGTGTTAGACTTGTCTTACAATCACATTGGGGTACTGGGTTATGGCTCATTTAGT
GGACTTCCCAACTTGAAAGCATTATATCTAACAGGAAACTCTTTGAAAGATCTCGGCTTCC
CTGCGTCTCTACCCAGCTTAGGTTACCTCATGTTGAATGATAATAAGTTGATGGCCTCGTC
GGTGGGTCTCTCATCGTTTGCCCGTAATGTTATGCATCTGAACATCCAGGACAACAGATTA
ACAAACCTGGAGGATGTTTACACCTTTCTGAATGAAATGAAACACCTTCAGTCTCTCTTCT
ACGGGGGAAACACAATCAGGTGGTGCACACTCAGTCGGCAAGTTTCAGGAATTGTTTTG
AATAATGTCCAAGTCCTTGATCTTCACAGCAGCTCACTGCAGTCTGTTTGGTCTCAGGGTA
AATGCCTGAATCTGTTTGACAATCTTGGACAAGTGACTGGTCTCAACTTGAGCTCCAACG
CACTACAATCTCTTCCTCAGGGCATTTTCAAGGGTCTCACCTCCATAGTGCAGATGGACCT
CTCATCCAACGCCTTGACTTATCTCCAGCCTGATGAACTACCCAAAAGTCTCAAAATACTC
AACCTCTCTAACAACTTCATAGCCTCCCCTGACCCTGCTGCTTTTCGTTCTCTCAGCCACC
TCAACCTAAATACCAACCGATTTCACTGCGATCACAACCTAAAGGGCTTCCTGACTTGGC
TGAACAAGACCAACGTAACCTTCCTGAGTCCCATTGAGGAGCTCAGATGTGAATTTCCTT
CCGGTTTCTATCATGTCTCTCTGCTAGATTACTCTGCTCAGGTCACAAAGCAGTAATTGTG
GGATTGTCTGGTTTG

Primer:
F: CGACCTCGGAGCCCAGAAT
R: CAAACCAGACAATCCCACAA

TLR2
Transcript ID：transcript5385_f2p0_3152
Annotation in NR：toll-like receptor 2 [Larimichthys crocea] KF318375.1

ATGTCCGATGAAGCCATTGTTAACTTCCTGGTGGTGTTTGATGGAGTGCCACTCACCTCCG
TTTGGGTGGATGGCATCATCCTGACTGGAGAGGGCAAGTGGGCACGAGCCAAATGGACC
AATCAGAAAAGCATTGATGAGTTCTTCTTACGAAACATTGTGGTGCTGGACGTCTGGAAG
TTTGTCTCATTGCTAGAGCTTGGATTTCTGCTGCATTATCCCAGGAAGGTGTCTGTCATAAA
CGGCAGGGTGTTTGTGATGCCCTGTCCTACATCCCGCCTGCTAGTGAACCTGCAGTACCTG
GACCTGTCCGACAACCTGCTGACTGAAATGACGCTGTCTGAGTCACTGTGTGACGGACAT
GGCACACTGAAGGACCTCCGGGTATTAAACATCAGTGGAAACGCTCTGAAGTCTTTGTCA
ACGATGAGCCGTCTGGTGGCAGGGCTCTCTAAACTCACCCACCTGGACATTAGCAGGAAT
GGATACAGCTCCATGCCCCAAGCCTGCTCCTGGCCCTCCACCCTCCGATACCTCAACATC
TCCAGGGCTAAACTTGAAACCATCACGCCCTGTCTACCCACAACTCTAGAGGTGCTGGAT
CTGAGCAACAATGATCTCAAAAGCTTAAATCTGTTCCTGCCTGCTCTGAGAGAACTCTAC
CTCTCTGGGAACAAGATTCAGAGTCTGCCTTCTGGATGGCTGCTTCCAAATCTGCAAATA
CTGACAATACAGTCAAACACCTTGAACATGTTTGGCCACTCAGACCTCCAGTCATACGAA
CGACTCCAGAACTTACAGGCGGGTCAGAACAAATTCGTCTGCTCCTGTGAATTTGTTGCT
TTCCTCCAGACGGACATTAAAGGTGAAAATGTGCAATTAACAGATGGCGAGGGGAGCTA
CATCTGCGACTCTCCTCTCTACCTGCAGGAGAAACCAGTGGGTCAAGTTCATCTCTCTGTT
GTGGACTGCCACCGGGTTTTGTTTGTGTCAGTGTGCTGTGGGGTGGCGCTATTTGTCGCCA
TTCTGTTGTGTATCCTGCTGTGGCGCCTCCACGTGTTGTGGTACCTAGAGATGACGTGGGC
ATGGCTCAAGGCCAAACGTAACTCCCGGCGAAGGCAGCGATGCAGAGAAAGAGAGGGC
TTGGAATCATTACTCTCCTTTGATGCCTTTGTGTCCTACAGTGAGCAGGATGCTAGCTGGGT
GGAAAACCTTTTGGTACCTGAGCTGGAAGAGCCAAGGGACAATGATGAAGAGTCTGTGA
ATCACCGGCCTTTGACTCTGTGCCTCCACAAGCGGGACTTCCTTCCAGGACACTGGATTG
TGGACAACATCATGAGTGCCATGGAACGCAGCCGGCGAACCGTCTTCGTCCTCTCTGAG
AATTTCGTCCAGTCCGACTGGTGCCGCTACGAGCTGGACTTCTCCCACTTCTGGCTCTTCG
ATGGGAACGCCGGCGGAGAGGCGATATTGATCCTCCTGGAACCGTTGTCCAAGGACGAC
ATCCCCAAACGCTTCTGCAAGCTGCGCAAACTTATGAGCTCCACCACCTACTTGGAGTGG
CCTCAGGAGGAGGAGAAGATTGGGGAGTTCTGGAGGAGTCTCCGCAAGGCTTTGAGAG
GAGAAGAGGAGGATTACTGA

Primer：
F:GGAGAAACCAGTGGGTCAAG
R:CAACAGAATGGCGACAAATAG

TCR
Transcript ID： transcript336_f2p0_5649
Annotation in NR: PREDICTED: transforming growth factor beta-2 [Larimichthys crocea]} XM_010752699.3

ATGAACCTGTGTATCGTCAGTCTCCTCCTAACTTTGGATTTAGCCACCGTGGCGCTCAGTT
TGTCCACATGCAGCACGCTGGACATGGATCAGTTCAAGAAGAAACGCATCGAGGCCATC
CGGGGGCAGATCCTGAGCAAACTGAAGCTCACCACCCCCCCTGAGGATTTTCCCGAGCC
CGAGGAGGTGTCCCGGGACATTGTCGCTATTTACAACAGCACCCGGGACCTGCTGCAGG
AGAAGGCGAACGAACGGGCGGCGACGTGCGAGCGGCAGCGGAGCGAGGAGGAGTATT
ACGCCAAGGAGGTGCACAAGATCGACATGCAGCCCTTCTATCCGTCAGAGAATGTTATCT
CTCCCACACACTTCAACCCTTACTTCAGGCGGCTGACGTTTGATGTGTCCTCCATGGAGA
AGAACGCCTCTAACCTGGTGAAGGCTGAGCTCAGGATCTTCCGTCTTCCAAACCACGGTG
CCCGAGTGTCTGAGCAGCGCATCGAGCTCTACCAGATTTTAGGGCACAAAGACCTGACAT
CCCCAACACAGCGATATATTGACAGCAAGGTGGTTCGAACACAAACAGAGGGAGAGTG
GCTGTCCTTCGACGTGACAGAGGCAGTGAGTGAATGGCTGAACCACAGAGATAGAAACA
GTGGATTCAAGATCAGCCTGCACTGCCCATGCTGCACATTTGTACCATCAAATAACTACAT
TATTCCCAACAAGAGTGAGGAGCTGGAGGCACGGTTTGCAGGTATTGATGACAGTTTTAT
CCATGGCGGTGATCTAAAGGTGTTTAAGAGGCGGCGGCACAGTGCACGGGCTCCACACC
TTCTCCTCATGCTGCTGCCTTCATACCGTCTAGAGTCCCAGTCCCAGTCCCAAGCCCCATC
CCAGTCCCAGCACAAGAACCATCGATCTAAGAGAGCCCTGGATGCTGCCTACTGCTCCA
GAAACGTTCAGGACAACTGCTGTTTACGGTCACTCTACATCGATTTTAAGAAGGACCTGG
GTTGGAGGTGGATCCATGAGCCAAAGGGCTATGAGGCCAACTTCTGTGCTGGGGCCTGTC
CATATCTGTGGAGCGCTGACACCCAGCATTCCAAGGTGTTAGGCCTATATAACACTATCAA
CCCTGAAGCATCAGCATCACCTTGCTGTGTCTCCCAGGATCTGGAGCCCCTTACCATCCTT
TACTACATCGGCAAGACCCCCAAAATAGAGCAGCTCTCAAATATGAAGGTCAAGTCCTGC
AAGTGCAGCTAG

Primer:
F: ATCTTCCGTCTTCCAAACCA
R: AGCCATTCACTCACTGCCTC

NF-kB
Transcript ID: transcript5497_f3p0_3102 (P65)
Annotation: Transcription factor p65 [Larimichthys crocea] XM_019270684.2

ATGGCGGATGTGTATGGATGGAGCATGACCACACTGAACCCAGTCCAAACAGCGAACCC
CTTCATAGAGATCATCGAGCAGCCGAAGCAGAGGGGCATGAGGTTTAGGTATAAATGTGA
GGGACGTTCAGCCGGCAGCATCCCCGGAGAGAAGAGCAACGACACCACCAAGACTCAC
CCAGCCATCAAGGTGCACAACTACACCGGCCCACTGCGTGTCCGCATCTCACTGGTGACC
AAGAATGCCCCACACAAGCCGCACCCCCATGAACTAGTCGGGAAAGACTGCAAACATG
GCTACTACGAGGCCGACCTGCAGGAGAGGAGAATACACAGTTTTCAGAACCTGGGCATA
CAGTGTGTCAAGAAGAAGGATGTGAATGAGGCCATCACTTGTAGGCTGCAGACCAATAA
CAACCCCTTCAACATTCCCGAGGCGAAGGTGTGGGAGGAGGAGTTTGACCTGAATTCGG
TTCGGCTTTGCTTCCAGGCCTCCATCACTCTGCCTACAGGGGAGCTGTTTCCTCTGGAGCC
CGTGGTATCGCAGCCCATCTATGACAACCGCGCCCCAAACACAGCCGAGCTGAAGATCT
GCCGCGTCAACCGCAACTCTGGAAGCTGCAAAGGAGGGGATGAAATCTTTCTGCTGTGT
GACAAAGTGCAAAAAGAGGACATCGAGGTGCGTTTCTTCCAGGACTCCTGGGAGGGTAA
GGGCACCTTCTCCCAGGCTGACGTCCACAGACAGGTGGCCATTGTGTTCCGCACGCCGCC
GTACCGTGACACTAACCTCTCCGAGCCCATTAGAGTCAAGATGCAGCTCCGCCGGCCCTC
TGATCGCGAGGTCAGCGAGCCGATGGACTTCCAGTACCTGCCTGCTGAGCCAGATGAATA
CAGGCTGAGTGAGAAGAGGAAGCGTACAGGGGACATGTTCATGAGCCTGAAGCTGGGG
CCCATGCTGTCTAGTGTGTCCATGCCACAAGACCGACGACACATAAGCCCGGCAAGGAG
AACAGTTACAGCCAAGCCTCCATCAATGAACACACAAGCTGCGGCTGTGGTGCCCCCTG
GTGCCAGTGGAGCGAAACCCCAGCCCTCCTACTCATACAATCAGCCGGGCCAGCTCTTCT
CAGTGCAGACAAAGGTAGAGGCCTCCCCCGTCTCTGCAAGCCAAACGTGGAGGAACAT
GGAGAGCCTGAGCCTGGGCAACCAGCCCAAAGCCACTCCAGTGTCCAGCTTCACTATGG
GCACCACCACCTCTACCACGATTTCCACTGTCAACCAGGACCTCTCCACTGTCAACCTGT
CAGACCTTCATGAATTCTTCCCCAACATGTCCTCGGGCATGGTCCAGGAGCCAGCAGCAC
CTCAGGGAAGCACGGTCCCCTCACAGATGAGCGGCTCCTTCCCCCTCCAGGGCTCTCAGT
TCCGGGTGGACCCACCGGTTGTGGATGATGATATCCCAGAGTTCCCTAGCTTTTCTGACGT
CCAGGCTCCTGGCACACTGGAAAACCTAAACATGGATGACTTTGTGGACCTTCTGAACCC
CACCCTCATGAGCGAGAGCGGAAACAGCACCTCAATGTTGGTTCAAGCCTCGTGCCAGC
AAGCTGCCAATTCCAGCTCCTCTACCGCTGTCCAGAACAGCGGGGCATGCCAGAACTCTT
CTGAACCCGCAAGCAACCCAGGAAGCACCTGGATGAATTACCCCAACAGCATTGTCAAC
TTGCTCCAGAATGAGGGCATGATGGACATGGCGCCCAACAACAACCACCACCCCCCCGT
GCTGGACGAGTTGGATGAGCTGATGTCCGCTGACGAGGATCGTCTTATTTCTATTCTTCAC
AGTGGAAACCAAGCTAGGTTTGTGTCAGGACACCCAACTTAA

Primer:
F: GTGGGAGGAGGAGTTTGA
R: CATAGATGGGCTGCGATA

MHC-II
Transcript ID: transcript21289_f21p0_1175 (MHC2β)
Annotation: MHC class II antigen beta chain [Miichthys miiuy] KC439427.1

ATGGATTCATCCTTCATCAGCTTCTCCCTCCTCTTCATCAGCCTCTACACAGCAGATGGATT
CATGAATTCCAGAGTGGCCCGTTGTGTTTTTAACTCGACTGATCTGAAGAACATCGAGCTC
ATCGACTCGTTCTATTTCAACAAAGATGAGATCATCAGGTTCAGCAGCAGTGTGGGAAAG
TTTGTTGGATTCACTGAGTTTGGTGTGAAGAACGCAGAGGCCTGGAACAAAGATGCTTCA
CGTATGGCTCAGATGAGAGCTCAGAAGGAGACATACTGTCTGAACCATGTTGAGAATGCC
TACCAGGCTGCTCTGGATAAGTCAGTTAAACCGTCTGTCAGGCTGCACTCTGTGACGCCC
CCTGGTGGTCACCATCCCTCCATGTTGGTCTGCAGCGTCTTCGACTTCTACCCCCAACACA
TCAAAGTGAGCTGGCTCAGAGACGGACAGGAAGTCACCTCTGATGTCACTTCCACTGAT
GAGCTGGCAGACGCTGATTGGTACTACCAGATCCACTCTCACCTGGAGTACACGCCCAGG
TCTGGAGAGAAGATCTCCTGTGTGGTGGAGCACGCCAGCCTGAAAGAACCTCTGATTACT
GACTGGGACCCGTCCATGCCTGAGTCTGAGAGAAACAAGATCGCCATCGGAGCCTCAGG
ACTGATCCTGGGTCTGATCTTGTCTCTGGCTGGATTCATCTACTACAAGAGGAAGGCCAG
AGGAAGGATTCTGGTTCCCAGTAACTGA

Primer:
F: ATCAGCTTCTCCCTCCTCTTC
R: AATCCAACAAACTTTCCCACA

TNF-*α*
Transcript ID: transcript13245_f2p0_2096
Annotation in NR: PREDICTED: tumor necrosis factor alpha-induced protein 8-like protein 2 [Larimichthys crocea] XP_010735433.1

ATGGAGGCCTTCAGCTCAAAGGAAATGGCCATGAAGGCGCAGAAGAAGATCCTCAGCTC
GATGGCCACCAAAAGCTCTGTCCAAATGTTCATTGACGACACCACCAGCGAGATCCTGG
ATGAACTGTACCGCGTCTCCAAAGAGTACTCAGGTAATAAATCAGAGGCCCAGAAAGTG
ATCAAGAATCTGATCAAGATTGCTGTGAAGATCGGCGTGCTGTTCAAGAACAACCGCTTT
AGCACAGAAGAACTGGGAGTGGCCCAAGATTTTAAGAAGAAACTGCACCAAGGGGCCA
TGACGGCTATCAGCTTCTACGAGGTGGACTTTACCTTTGACAAGGCTGTGATGGAGGAAC
TCCTGACCAACTGCAGGGATCTGCTGCTGAAGCTGGTCAACAACCACCTCACCCCTAAAT
CCCACGGTCGCATCAACCACGTCTTCAACCATTACTCCGACCCAGAGCTCCTGACCAAAC
TGTACGATCCCAGTGGCCCATTCCGACCCCACCTTACTAAGATCTGCAAAGGACTTGACA
AACTGGTAGAGGAGGGGACAATATGA

Primer:
F: ATCGGCGTGCTGTTCAA
R: GCGACCGTGGGATTTAG

IL-1
Transcript ID: transcript10446_f2p0_2415
Annotation in NR: IL-1-related receptor [Collichthys lucidus] XP_019109540.1

ATGGCTGTGATTTTAGTCGCTGTCTTACTGGTGTGCACTGTTGAATGGGACAAGAGTTTATT
CGCAGCCGCTCAGTCCTGTGTGGATGAGAGCAGGTTTAAGGAGCAGGTGCTCTATGTGGG
GCAGCAGTGGCCTCCATATCGGCTGAACTGCCCTCTGGAGCTTGTCCAGTCCCAGCTCAA
GTTGATCTGGCAGAAGGACTGTCAGCAGCTCCGCACCCAGGAAGGGAAGGCCTACGTGG
AGTTTGCCAGCCTCAGCTTCCAAGACCAAGGGAATTACACATGTGTGCAACAGGGCAAC
AGCACAACCTCATTCACAGTGCGTCTCATAGTTAAAGAGTCCCAGTGCTCCAAAGCCCCA
GAGTTTAAACCAAATGGGGGTCCGACCAGACTCTGGAGGAATGTGGGATCCACAGTGAT
ACTGAACTGCTCTGCTCTCCTCTTCTGGGACCCAAAAGAGAAGCAATGTGACACCACGCT
GCAGTGGAGTAAAGATAGCCAACCCCTCACCAATCACACACTTTACAAGCAGAATATCTC
CTCATGGTCTCCTGTTGCCGACCAGCTGATGGTAAACAGTCTGCTGGAGATCACCCTCAG
GGAGCTGGACGATTTTGGGCTCTACTGTTGTGCAGTGAGGAACATTTCCTCTGAGTTTAGC
CTACACGCTTCAAGCGGCCCCAACCATACGGCTGCTGTTATTGCAGCCATTATCCTCCTCC
TGTTACTGAGTATAGCTGCTGTCGTGTACTCCAGGTGTCACCTAAATATCAAACTGTGGTA
CAGAAACACCTATGGAGACTATGAACTCAGTGATGGTAAATTATATGATGCCTATATCTCC
TACGTGAACAATGACCATGACAGGAAGTTTGTCAACTTTATTCTCAAACCTCACCTGGAG
AATAAAAATGCATACAAGGTTCATCTCAATGATAACGAAATCCTACCTGGCTCAGAACCT
TCTGCGGAGCTGCTCATGAACATAAGTCGTTCTCGGCGTCTGATTGTGGTGCTTTCTTACG
CTTACCTTGAGCAGGACTGGTGCTGCAACAACTTCAGACAGGGCCTTCTGCACTTGTTGG
AATTATGTAAGAGTCCCATCCTCATCGTGTTGGAGGGTCAGTCCAAACGCATGAGGCCTG
AGATCAAGCAGCAGCTCAGTGAACACCAGCACCGCCTCACTGTACTTACCTGGAGGTAC
AACTCCGTGACTCCCTCCTCAGTCTTCTGGAAGGAGCTGGCCTTAGCGATGCCTCGCAAA
GTCGTCTTTCGCAGTGAGTCTGTAGGTGACCCTCAGACTATGCTACAAGATGACAAGGAC
CCCATGCTGACCCTCAACCCCGACTACCTAGACTGCCGCTCAGACACAGACCCTGCTGG
AGATCTAGGGCTTCGCCTACCTGTGTACAAGGCCATGGCCTGTAAAGCCCCTGTCCTCCCT
GCTGCTCCCATGACTGCAGCTGAGCCCAAACCTGTGGACATCGACGTGTCGGACCTGGG
CTCACGCAACTATGGCGCCCGCTCAGACTTCTACTGCCTGGTCACTGAGGACATATGA

Primer:
F: GTTCTCGGCGTCTGATTGTG
R: GTGAGGCGGTGCTGGTGTTC

IL-6

Transcript ID: transcript2470_f2p0_3891

Annotation: Interleukin-6 receptor subunit beta [Larimichthys crocea] XP_010747226.1

ATGGAGTCTGCCAGACTGTTCCAGCTGTTGCTGTTAGCCTGCCTAGCCTTTGCTCTACCTG
CCCGAGGAGAAAATCTCAGTCATTTGGTGATAGTGCCACAGTCTCCAGAGCTTGAGATTG
GAACTAACTTTATGGCTACATGCATGATCTTCAACACATCTGAAGTTACAGCAGACGACC
TCTACTGGAAGCTGTCGAAGACTATTATACCCAAAGAGCAATACACAAAGATCAACAGCT
CAGCTCTCAATGTCACTATCCCCATCACCACTGAGAGCTCTGAATTGCTGTATTGCTTCTG
CAAGGGAAAGTCAAACTATGTTTACCTGAATCCAGGCAAATTTGTGCATGGGATCTTCCT
GAAGAAAGGCTATCGCCCAGGGAAGCCTGAGAATTTTTCCTGTATGGCAGTTCAGGATAA
AACAGTCATCTCTGAAATCATCATGTGTAAGTGGGACCCAGTAGGACGTCAAACCACGG
ATGTCCCTACAACGTACACACTGAATGTTAAAGTCACCCCCAGTGATATCAAGAATAAAT
CAACAAGTGAAAATAGTATCCAAGTGGCTCTGGAGGTTTTCCCTTATCATATGACTATGGA
GATCTGGGTGGAGGCACACAATAAACTGGGAAGAATAGAGTCTGAGCATCTGAAAGAA
GATGCTGGCTGGTTTGTGAAAACGAACCCTCCATCAAATGTCAAAATCATTTCAGAGAAG
AGTTTTCCCATCTCCCTTCTCATAAACTGGACTCATCCAATAGCTGAAGAATATCTGAAAT
TAATATATAAAATCAGATTCTGCCCACAAGGATCTCAAAGTTGGACTGATGTACCTCTTGT
GGACACTGCCAAGCACATACAGTCCTTCAGACTACAGAACCTTCAGCCAGACACTGTTTA
TATTGTTCAGGTGTCCTGCAAAAATGCCAGACTTGGCCATGGTTACTGGAGCAACTGGAG
TACCAATGCAACCAGTAGAACACCAGAGGACAAACCTTCCAGTAAACCAGAGTTATGGA
AATTCATTTCTGATGGTGACAACATGAACGAACAACAAATGCAGATTATTTGTAAGGATC
CTGTGTTCGCCAACGGAAAGATAAACAGATTTGAAATGAAGATTCAAGACCTAACAGAC
AAAGGCAAGAATGGGAGTTGGACATGGGAGAGTATTCCAGTCAACAGGTCAGAGGCCA
ACAGCAGGTCTAGTCAGCACATCCTGAAGGAGATTCACCTGGCTGACAAGAAGTCCATC
AAAGTGTATGTCAGCGCCTTTAATTCTGCAGGAAAATCTCCTGAGGCAGTGTTAGTCATCC
CTGAAAAAGAGCTTCCCCCAGTGGAAGAACTGAAGGTGTGGCCCCATGAAGGCAAGCT
GCAGCTGGAATGGAAACCCCCCAAAGGTCGCTCTGTCCCAGAGTATGTGGTGGAGTGGA
TTAGTGGTGATCAGAGAGGCTGGCAGAGGGAAAACAGAAACACCAGACACACTTTCATT
AAAGGCACCCTTGAGAAGTTTGTTTGCTACAAAGTATCTGTGTATCCAATCTACTCTAATG
GATATATTGGCAAGCCGGCAAGTCAAGAAGCCTTTCTGGAGCAAGGAGTTCCATCAGATG
GCCCGACCATTACACTGATTGCTAAAGGATCTAACAATGCAGAGCTGGAGTGGTCAGAG
ATTCCCCAACACATCCGACGAGGCTTCATCACACACTACACGATATACTATAAAAGCGGG
ACTGAAACACATGCCATAAAAGTGCCAGCTAACGTCACCTCATACGTTCTGAGGTCATTG
TCACAGAACACCAAATATGACACTTGGATCGAAGTTTCAACCATCAAAGGGTCAGCCAA
AGGCTATAATCACTCATTCACTACTCTGAAATATGCACCCGGAGAGATCGAGGGCATCGT
GGTAGGAGTGTGCCTTGGCTTTCTCTTTATTGTCCTCATGACCATGCTGCTGTGTATCTACA
AAAAAGATGTGATCAAGAAGAATTTCTGGCCCTGGATTCCAAACCCTGGAGAGAGCACC
ATTGGAAACTGGTCTCCTGATTATCCTCTTAAAGCAGAGACACCAAAGGAAAACTGTGTA
TCTGGCATCAGTGTGCTTGATGTGGATGTGTGTGATGGAAAGTGTGTGTTTGAGGAGGAC
AAGGCCAGTCTTCCTCTAAAGAAGGACAAGTATCTGTCTGAGGAGCACAGCAGCGGCAT
TGGTGGCTCCTCTTGCATGTCTTCACCTCGCCAGAGTGTGTCTGACAGCGATGAGGGCGG
CGACATGGCTGACACCACAGCAAGCACTGTTCAGTATTCGTCGGTGGTGGCCTCCAATGG
TTACAAGGGTCAGACCCCAAGCTCCCAACCTCAGCAGGCCATTTTTTCACGGTCTGAGTC

CACGCAGCCTCTTCTGGATTCTGAGGAGAACCCAGACATGTTGCTGCAAGAGGGCAGCA
GACAGTCCCAGCGTTTCCCTCGACAGGCCTGCTTTACACACACTGCACGGAACAAAAAC
AGCACCGACCCTGCTGACTCCAACCAGCTGGAGATGGAGCAGCAGGAGATGGTGGAGC
CTTTGGACTTCTGTCCTTTGGAAGAGGACTCTGAGCAGACAACACCTGCTGACAGTCATT
CAGCTGACTGGCTGTCAGAAGCACCAATCTCCAGCTACATGCCTCAGCTGGGTGGTTACA
GGCCACAGTGA

Primer:
F: GCCCAGGGAAGCCTGAGAAT
R: GGGACATCCGTGGTTTGACG

BH3
Transcript ID： transcript400_f3p0_5509
Annotation in NR: bcl-2-like protein 11 [Larimichthys crocea] XM_010739224.3

ATGCATCATCTATCCAGACCACCAAACCGGTCCGATGGCTCGACCGCAGTAGCGGGAAC
ACAAGGGAGCGGAGGAGATCCATCACCCGCCGGTGCTGCTGGAGCCTCAGCGCAAACC
TCCCGTTTAGACAACGGCGGCGAGCGGAGCTTTGGCCACAGCAACTCCACCGGAGGAGG
AGAGCCGGACTCGCCGTCCTGGTACAGAACCAAACCCATCTCCCCTCTCGACAACAGCC
TAGGCGTGTTTCAGACGAGGACGATATTCCACCTCCCACGCCGCGCCTCCAGTGGATATTT
CTCCTCGGACGGCGACTCGCTGCCAAGCTCTCCGCACACCCCGAGACCAGTGACGGCTG
ATAAAGCCACGCAGACTCCGAGCCTCACCGGCCAGGTGGTGCAACACGCCGTACTGCGC
ATGACAGAGGCGCCCGGCGGAGAACCGGGGACGCACCAGCAGCACGGACATTCTCCCA
GCCCCTCTAGCATGCGGCCACGAAACGCAGCCGGGGACATGCAGACGGAGGTTATCGGA
CGAGAGCTCCGACGCATTGGAGATGATTTCAACAGACTTCTTCTCTTAAGGGGGATGGCC
AACCCAAGACGGGTTGTGATCCATCCGAACCCACTGCCGCACATCCACCAGGAACCCGC
CATGCTGCTCTGCATGGGCCTCCTGCTCCTTCTGATTGGACGGATAATCTACTTGCAGGGC
AATATGGACAGCCCAGACCACTCTCAGGTTTAG

Primer：
F: CCCAAGACGGGTTGTGAT
R: CCATATTGCCCTGCAAGTAG

Casp8
Transcript ID: transcript14011_f8p0_1992
Annotation in NR: caspase 8 [Totoaba macdonaldi] KY689036.1
ATGGATAGGGTGATGCTGTCCCGAATAGATGAGGAGCTTGAGTCCTCGGAGGTGGCCGCT
CTCTGCTTCTTGTGTCACGATGTCGTCAACAGGAAGCGCCTCGAAGGGGTCATGGATGCA
AAAGCGCTGTTCTTGAGACTAGAAGAAAGAGGTCTGCTGGATAACCACATCTTCCTTTCT
CGGCTGATTAACACCATCCGGCGAGCAGATCTCCTCAGCCTTCTGGAGACAGACAGCAG
GCAACCAGTAGAAACTGATGCAAATCCTTTGCTGTCAGAATACAGGGTGATGCTGTACCG
TATATATGAGGACATGACTCAGGAAAACCTTGACAAGATGAAGTTTCTGTTGAGTAGCAA
GCTGGGCAGAAGACAGATTGAGGCATGCAATACAGCCCTGGATGTGTTTGCTGAGATGG
AAAAGGCTTGTTTACTGTCAAAAGACAAACTTGATGAGCTGCTTGCAGTACTGCAAGAGT
TTGATCAACAGCTGGCATCGACTGTACGGCTCCACATGCAAAATGAGTGCAGAATGAGG
CCAATCCCACCGGCTCATGTCAGCATGGATGACCAGAGGGTCATCAGAATCTGTCAGGA
AAGACCACAACCTGAGACACAGCCAAGCGATGCAGGACAGACTGTTTACACTGATGCA
GAACTAAACTTGTCTCCTTCAGATCAGACAGAGTACTACGCCCTGATACATAACCCTCGT
GGTCAGTGTGTGGTTTTCAACAATGAGAAATTCCAGAATGTAAACCTGAAAGATAGAGG
AGGGACTCAGCAGGATGAAAAGGTTCTGTGTAAAGTCTTCAACCGCCTTGGCTTCACTGT
GGAGGTGCACAACGACTTGACTGCAGCAGAAATAAGACACAAAATAAAACAGCTGGGC
AGTAGGAATTTTTTGAATGATGATGTCTTGGTGGTATGCGTGCTTTCCCATGGAGAAAAGG
GATGCATCTTTGGGACTGATGAGAAGCCGGTGGACCTGCGAGAACTGACAGAGCCCTTT
ACGAGTGGGAATGCTCCCACCTTGGCAGGGAAGCCCAAGCTGTTCTTCATCCAAGCGTGT
CAGGGAAACAACTACCAGATGGGAGCTGTGCCATGTCCACCGAGGCCAAGACAGGAGC
TGGACAACAGGGAGAACCGACTGGAGGAAGATGCAGGTCCTGTGCGTGGCGAGACGAT
ACCTTCGGATGCTGACTTCCTGCTGGGCATGGCCACCGTGCAGGAGTGCAAATCGTTTCG
AAACACTTCCACAGGCTCCATCTACATCCAGCAGCTGTGCAGCCAGCTGATGAAGTCAG
CACAAAGCCTGGAAAATGATGATATACTCACTGTCCTGACACGTGTGAACAGAGAGGTC
AGCAAAGGAGAATATTTAAGCTACAAACAGATGCCAGAGCCCAAGTACACCCTCACCAA
GAAGCTCGTCTTCAAATGTGTATGA

Primer:
F: GGAGAACCGACTGGAGGAA
R: TGTAGATGGAGCCTGTGGAAG

Bcl2
Transcript ID： transcript10299_f7p0_2378
Annotation： apoptosis regulator Bcl-2 [Larimichthys crocea] XM_010729997.2

ATGGCGAACGAGCGTAACCGCAGCATTGTGGAAAGTATATCTGCCATAAACTCTCCAAAC
AGGGCTACGTGTGGGGGTTTGACGATGTCCGGGATGAAGATGCTGCTAATAACGGGTCAA
TAGTTGCTCCTCCACCGACTTTAGTCCGCCGGTGCCGTGAGGCCAGCACCGGGCCTGACA
CCGAGAGCATCCCCCACCTCTGCAAACGTCTCCCCCAGTCCGACCCGCACGCCGCCATC
CACAGAGTCCTGCGCGAGGCTGGAGATGAACTTGAAAGACTGTACCAGCCAGACTTCAC
GGAGATGTCACGGCAGCTGTATCTCACCTCCACCACGGCGCAGAGGAGATTCGCCGAGG
TGATAGACGAACTGTTCCGGGACGGGGTGAACTGGGGCCGGATTATCGCTTTCTTCGAGT
TCGGGGGCACGGTGTGCGTGGAGTGCGTGGCCAAGGAGGAGATGGCACCGCAGGTGGA
CAACATCGCGGAGTGGATGACGGAGTATTTAAATGGACCTCTGAACAGCTGGATACAAG
ATAACGGGGGATGGGATGCCTTTGTAGAGCTGTATGACAGACAGAGGGACTCCGTCTTCA
GTTGCTCCTGGCCCTCCATTAAGACGGTCTTCGGTATGGCTGCGCTCGGGGCAGCCAGCC
TCACCATCGGGGCATACCTTACACAGAAGTGA

Primer：
F: CGGGTCAATAGTTGCTCCTC
R: CCGTGGTGGAGGTGAGATAC

Bax
Transcript ID: transcript6117_f4p0_2977
Annotation in NR: apoptosis regulator BAX [Larimichthys crocea] XM_010749348.3

ATGGCTGACAGTCGAGAAGAGGAGAAAAAGGCAGACCGGGAGCCTGAAGGCGCCACG
GGTGGGGAAGATGTCATCGATGATCCCATTCTGGAGCAAGGAGCAGTAGTCCTCAGAGG
GTATGTGATTGAACGTATAAACACAGCGGAGCCCAGTCTGCATGTCTCCTCTGAAGACCT
GGGAGGAAGGCCAAACGAGCAACAGGATCCACAAATCAAAGAGGTGGTGGAACATCTG
CTCAAGATTGCAGAAGAGCTGAACAGGAACGCCGAGCTCCAACAATTGATCAACCAGGT
TCAGAGCAACTGTGCTCAAGACATCTTCATGAAGGTGGCCAGGAGCATCTTTGCTGATGG
CATCAACTGGGGCCGAGTGGTGGCTCTCTTCCATCTGGCCTACAGACTCATATACAAGGC
ACTGACCACCAACCACCTAGAGAACATCAGAACAGTCATCAGGTGGGTTCTTCAGGTCA
TCAGAGAGCAGCTCTACTCCTGGATTGTACAGCAGGGAGGCTGGGAAGGGGTGATCCGA
GGTTTTTCTCGGTGGAGGACAGTAGCTGTAGTAGCATCAATAGTATTGGTGGCAACTATTG
TTTACTACAGGAAGACACGCTGA

Primer:
F: GAGGTGGTGGAACATCTGCT
R: TTGGTGGTCAGTGCCTTGTA

Casp3
Transcript ID: transcript10942_f11p0_2044
Annotation: caspase 3 [Miichthys miiuy] KF738809.1

ATGTCGGCTAACGGACCCGGACCTGGAGGAGACTGCACAGACGCGAGGAGAGGCGATG
GACAAGAGTCGGAGCCGTCTTCGTCTGCTGCCGCCACCAAGCCGGGCTCCCACAGCTTC
AGATACAGCCTGAACTATCCCTGCATTGGCCAGTGCATCATCATCAACAACAAGAACTTT
GACAGGAGTACAGGCATGAATCAACGAAATGGTACAGATGTAGATGCAGCCAACGCGAT
GAAAGTGTTTGCAAAGTTGGGTTATAAAGCGAAGGTTTACAATGACCAGACAGTCGACC
AGATGAGACAGGTTTTAACTTCTGTTGCGAAGGAAGATCACAGCTGCTACGCCTCGTTTG
TCTGTGTTCTGTTGAGTCATGGAGATGAGGGAGTGTTCTTTGGTACGGATGGCTCTATAGA
GCTGAAGTACCTAACATCACTGTTTCGAGGCGATCGCTGCAAATCACTGGTGGGAAAGCC
CAAACTCTTCTTCATCCAGGCTTGCCGAGGCACAGATCTGGATGCAGGCATTGAAACCGA
CAGTCCTGATGATGGCACTACAAAGATCCCTGTGGAAGCTGACTTCCTCTATGCTTTCTCC
ACAGCCCCAGGTTACTACTCATGGAGGAATACTATGACCGGATCCTGGTTCATGCAGTCA
CTGTGTGATATGATCAGCAAGTATGGAAAAGAAGTGGAGCTCCAGCACATCATGACACG
AGTGAACCACAAGGTGGCAGTAGAGTTTGAGTCTGTCTCCCACTCACCAGGCTTTCATGC
AAAGAAACAAATCCCATGCATTGTGTCAATGCTGACCAAAGAGATGTATTTTTCTCCTTG
A

Primer:
F: CTGCTACGCCTCGTTTGTCT
R: TCAGCTTCCACAGGGATCTT

β-actin
Transcript ID: transcript11384_f3p0_2155
Annotation in NR: actin, beta [Coregonus maraena] HE984309.1

ATGAAAATCGCCCGCACTGGTTTGTTTGACAAACGGATTCCGGTATGTTGCAAAAGCCGG
ATTTCGCCGGGAGAACGGACCGCCCCCTCGTGCTGTCTTTCCCCATCCATCGTCGGTCGCC
CCCAGGCAATCAGGGGCGTGGATGGTTTGGTATGGGGCCCAGAAAGGGACAGCTATGTT
TGGTGATGAAGCCCAGAGCAAGAGAGGTATCCTGACCCCTGAAAGTACCCCATCGAGCA
CGGTATTGTGGACCAAACTGGGATGACATGGAGAAGATCTGGCAATCACACCTTCTACAA
ACGAGCTCAGGAGTTTGCCCCCTGAGGGAGCAACCCCCGTCCTGCTCACAGAGGGCCCC
CCCTGAAACCCCAAAAGCCAAACAGGGAGAAGATGACCCCAGATTCATGTTTCGAGACC
TTTCAAAACACCCCTGCCATGTTACGTTTTGCCAATCCCAGGGCTGTTGCTGTCCCCTGTA
TGCCTCTGGTCGTACCACTGGTATCGTCCATGGGACTCCGGTGATTGGTGTGGACCCCACA
CAGTGGCCCCATCTACGAGGGTTTACGCCCCTGCCCCCACGCCATCCTGCGTCTGGGACT
TGGGCCGGCCCGCGAACCTCACAGACTACCTCATGAAGATTCCTGGACAGAGCGTGGGC
TACTCCTTCACCACCCACAGCCGAGAGGGGAAAAATCGTGCGTGGACATCAAGGAGAA
AGCTGTTGCTACGTCGCCCCTGGGACCTTCGAGCAGGAGATGGGGCCACTGCTTGCCTCC
TCCTCCTCCCTGGAGAAAGAAGCTATGAGCTGCCCTGACGGGACAGGTTCATCACCATTG
GCAATGAGAGGTTTCCGTTTGCCCCAGAGGGGCCCTCTTCCAGCCTTTCCTTCCTCGGTAT
GGGAAATCTTGCGGAAATCCCACGAGACCACCTACAAACAGCAATCATGAAAGTGCGGA
CGTCGACATCCGTAAGGACCTGTATGCCAAACACCGTGCTGTCTGGAGGTACCACCATGT
TACCCCGGCAATCGCCGACAGGATGCAGAAGGAGATCCACAGCCCCTGGGCCCCCATCC
CACCATGAAAGATCAAGATCATTGCCCCCACCTGAGCGTAAAATACTCTGTCCTGGATCG
GAGGGCTCCATCCCTGGGCCCTCTCTGTCCCACCTTCCAGCAGATGTGGATCAGCAAAGC
AGGAGTACGATGAGTCCGGCCCCCTCCATCGTCCCACCGCAAAATGCTTTCTAAAACAGG
GACTGTTTTCCTCCTACCCCCCTCCCCAAACCAAAATGCCCCAAACAACTTTCAGCTCTGT
GCAAAAAACAAACCACACACCACCACACATTTTCTCATACACACTCAGGCGCAGAAGCC
TAGATGGAACCAAACTCAATTGGCATGGCTTCAGTTATTTTTTTGGCGCTTGGACTCAGGA
TTTTTTAAAAAAAAAACTGGAAACGATGAAGGAGACAGTAAATGTTTTTTTGGCTAGGTT
TTAA

Primer:
F: AAATCGTGCGTGGACATCA
R: CCGTCAGGGCAGCTCATAG
